# Federated Reinforcement Learning-Based Dynamic Resource Allocation and Task Scheduling in Edge for IoT Applications

**DOI:** 10.3390/s25072197

**Published:** 2025-03-30

**Authors:** Saroj Mali, Feng Zeng, Deepak Adhikari, Inam Ullah, Mahmoud Ahmad Al-Khasawneh, Osama Alfarraj, Fahad Alblehai

**Affiliations:** 1 School of Computer Science and Engineering, Central South University, Changsha 410083, China; malisaroj@csu.edu.cn; 2 School of Information and Software Engineering, University of Electronic Science and Technology of China, Chengdu 610054, China; deepakadhikari@uestc.edu.cn; 3 Department of Computer Engineering, Gachon University, Seongnam 13120, Republic of Korea; 4 Hourani Center for Applied Science Research Center, Al-Ahliyya Amman University, Amman 19328, Jordan; mahmoudalkhasawneh@outlook.com; 5 School of Computing, Skyline University College, University City Sharjah, Sharjah 1797, United Arab Emirates; 6 Computer Science Department, Community College, King Saud University, Riyadh 11437, Saudi Arabia; oalfarraj@ksu.edu.sa (O.A.); falblehi@ksu.edu.sa (F.A.)

**Keywords:** federated reinforcement learning, federated learning, reinforcement learning, edge computing, internet of things

## Abstract

Using Google cluster traces, the research presents a task offloading algorithm and a hybrid forecasting model that unites Bidirectional Long Short-Term Memory (BiLSTM) with Gated Recurrent Unit (GRU) layers along an attention mechanism. This model predicts resource usage for flexible task scheduling in Internet of Things (IoT) applications based on edge computing. The suggested algorithm improves task distribution to boost performance and reduce energy consumption. The system’s design includes collecting data, fusing and preparing it for use, training models, and performing simulations with EdgeSimPy. Experimental outcomes show that the method we suggest is better than those used in best-fit, first-fit, and worst-fit basic algorithms. It maintains power stability usage among edge servers while surpassing old-fashioned heuristic techniques. Moreover, we also propose the Deep Deterministic Policy Gradient (D4PG) based on a Federated Learning algorithm for adjusting the participation of dynamic user equipment (UE) according to resource availability and data distribution. This algorithm is compared to DQN, DDQN, Dueling DQN, and Dueling DDQN models using Non-IID EMNIST, IID EMNIST datasets, and with the Crop Prediction dataset. Results indicate that the proposed D4PG method achieves superior performance, with an accuracy of 92.86% on the Crop Prediction dataset, outperforming alternative models. On the Non-IID EMNIST dataset, the proposed approach achieves an F1-score of 0.9192, demonstrating better efficiency and fairness in model updates while preserving privacy. Similarly, on the IID EMNIST dataset, the proposed D4PG model attains an F1-score of 0.82 and an accuracy of 82%, surpassing other Reinforcement Learning-based approaches. Additionally, for edge server power consumption, the hybrid offloading algorithm reduces fluctuations compared to existing methods, ensuring more stable energy usage across edge nodes. This corroborates that the proposed method can preserve privacy by handling issues related to fairness in model updates and improving efficiency better than state-of-the-art alternatives.

## 1. Introduction

The Internet of Things (IoT) is connected with various sensing devices that generate a deluge of sensing data from various IoT applications, such as agriculture and food industries. IoT applications in agriculture as a whole rely on interconnected devices such as sensor networks, drones, and automated machines. These devices generate an enormous volume of heterogeneous data from diverse sources, including soil moisture sensors, weather stations, satellite imagery, and farm machinery. However, these data are often redundant, noisy, or inconsistent, limiting their utility for real-time decision-making [1,2]. To address these challenges, multi-source information fusion has become a critical methodology for integrating, processing, and analyzing data from various sources to provide comprehensive, accurate, and actionable insights.

Multi-source information fusion has become a critical methodology for integrating, processing, and analyzing heterogeneous data from diverse IoT applications, enabling enhanced accuracy, robustness, and extended applications in real-time decision-making for agriculture. For example, fusion techniques such as Bayesian inference, neural networks, and fuzzy logic have shown significant improvements in optimizing resource allocation and scheduling tasks [3]. The integration of information fusion and edge computing further minimizes energy consumption and latency while maintaining data privacy, transforming traditional Agri-IoT systems into efficient, resilient, and sustainable frameworks [4].

Information fusion in IoT applications enables three key improvements:

1. Enhanced Accuracy: By merging information from varied origins, fusion decreases the impact of mistakes or disturbances from single data flows. For instance, combining soil moisture details with weather predictions and drone images grants a more exact interpretation of crop water necessities.
2. Improved robustness: The combination of information reduces reliance on just one data source. This makes sure that the system continues to work correctly and can be trusted, even if some sensors do not operate or generate incorrect data. Having this strength is very important in agricultural settings that are always changing and cannot be predicted easily.
3. Extended applications: Through the combination of different datasets, information fusion opens up new potentials. These include the detection of pests in real time, exact irrigation methods, and improved schedules for harvesting.

These improvements in combining information directly impact critical operations in agriculture IoT like resource allocation and scheduling tasks. Resource allocation makes sure that the computational resources, such as processing units, storage, and memory, are used well. For example, bringing together merged data from drones and sensors placed on the ground assists in dividing necessities like water or fertilizer exactly at the place and time when they are required. In the same way, organizing tasks includes managing farming actions like planting, watering crops, controlling pests, and gathering crops based on actual conditions. Through studying combined data, intelligent planning systems can improve these activities. Aspects such as soil state, stages of crop growth or development, availability of machinery, and forecasting about weather are considered.

The use of information fusion is very important to deal with the natural inefficiencies and privacy issues that are present in centralized Agri-IoT systems. These centralized systems frequently have delays, consume a lot of energy, and can be weak against data breaches—all these problems seriously affect the success of smart farming efforts. To extend beyond these barriers, this study brings together edge computing and information fusion along with machine learning algorithms to improve the performance, security, and sustainability of IoT systems. Edge computing handles data near its origin, reducing latency, energy consumption, and the risk of data leakage during transmission. Together with information fusion, edge computing makes it possible to conduct analysis and make decisions in real time, even in environments with limited connectivity.

IoT devices generate tremendous amounts of data that need significant computing power for analysis purposes—this kind of effective resource allocation is crucial in maintaining operational efficiency and cost-effectiveness, adapting to fluctuating environmental conditions and market dynamics to enhance productivity. Dynamic resource allocation in IoT application systems encompasses the efficient utilization of computational resources such as processing power, memory, and storage [5]. It also includes managing physical resources, including sensing devices, monitoring equipment, machinery, and other related resources.

IoT in agriculture, also known as Agriculture 4.0 [6], uses sensor networks, drones, and automated machines to gather tremendous amounts of data for optimizing agricultural outcomes [7]. Efficient management of computational and physical resources is critical for the effective operation of this technical framework. Dynamic resource allocation and task scheduling play pivotal roles in this optimization process [8]. Task scheduling is equally crucial, involving the coordination of various farm activities—planting, irrigation, pest control, and harvesting—taking into account real-time factors such as weather conditions, soil moisture levels, equipment availability, and crop growth stages. Smart task-scheduling algorithms dynamically adjust to optimize resource utilization and yields, ensuring timely and efficient farm operations [9].

This research is driven by the need to tackle the privacy issues, inefficiency, and delay problems linked with centralized Agri-IoT systems [8]. By tackling the challenges associated with dynamic resource allocation and task scheduling in edge-based IoT environments using a hybrid form of Bidirectional Long Short-Term Memory (BiLSTM) [10] and Gated Recurrent Unit (GRU) [11] for task scheduling along the attention mechanism, and Federated Reinforcement Learning for resource allocation, this research contributes to the development of more efficient, scalable, and adaptive decision-making frameworks. The integrated use of data fusion in edge computing with novel hybrid algorithms to predict and optimize dynamic task scheduling helps to reduce energy consumption. Similarly, Federated Learning integrated with Reinforcement Learning helps to augment the privacy preservation of the data, dynamic resource consumption, and user equipment selection for efficient operation and decision-making. In summary, our contributions are as follows:

1. Novel Hybrid Prediction Model: We present a novel hybrid model that merges Bidirectional Long Short-Term Memory (BiLSTM) and Gated Recurrent Unit (GRU) layers with an attention mechanism for projecting resource use in IoT applications, which can be used for predicting dynamic task scheduling.
2. Proposition of an Edge-Centric Offloading Algorithm: Our algorithm optimizes task allocation, enhances performance, and reduces energy consumption by utilizing predicted resource demands.
3. Novel Federated Learning Algorithm: We present a novel Federated Learning algorithm utilizing Deep Deterministic Policy Gradient (D4PG) for dynamic user equipment (UE) participation based on resource availability, tested against DQN, DDQN, Dueling DQN, and Dueling DDQN models using the EMNIST dataset, demonstrating its effectiveness in addressing model update fairness and enhancing overall efficiency.

The rest of the paper is structured as follows: Section 2 reviews existing approaches for resource management in Federated and Reinforcement Learning contexts, Section 3 presents the problem formulation, Section 4 presents the system design and details the proposed methodology, and Section 5 discusses the experimental results. Finally, Section 6 concludes the study and outlines potential future research directions.

## 2. Related Works

Paper [12] proposes an optimized system model for task offloading in 5G heterogeneous Mobile Edge Computing (MEC) networks, focusing on balancing processing time and energy consumption through a multitasking framework (MTO) and a Multitasking Evolutionary Algorithm (METOA). Paper [13] integrates IoT devices, MEC, and cloud layers, distinguishing between ordinary and VIP users. It employs differentiated services and security levels, with tasks offloaded based on real-time requirements and security needs across public and private nodes.

Edge computing and Agricultural IoT (Agri-IoT) have undergone notable progress in recent years to tackle distinct problems like instant decision-making, limited resources, and data protection [14]. This exploration investigates essential research and movements that are sculpting the world of edge computing in Agri-IoT.

In precision agriculture, edge computing was presented in [15]. It highlighted the importance of managing data locally and making decisions in real time. The method applied in [16] used pre-trained models based on a convolutional neural network (CNN) to detect plant sicknesses, adjusting hyperparameters for good functioning. A custom edge fog-cloud structure for IoT agriculture applications was suggested in [17], handling intermittent internet connections with a unique framework of edge computing [18]. The worries over security were tackled in [19]. The authors put forward a structure that brings together software-defined networking, blockchain technology, and fog computing to enhance Agri-IoT security.

Edge and fog node architectures in Agricultural IoT were studied in [20], and a five-layer hybrid IoT system architecture was introduced in [21] to improve deployment options. The idea of ad hoc networks using mobile devices for edge computing was put forward in [18], while the combination of edge computing with blockchain, AI, and virtual/augmented reality in Agri-IoT was explored in [22].

Though there is notable advancement as shown in [15,16,17], study [19] pointed out ongoing restrictions. Essential elements like dynamic resource allocation and task scheduling for improving Agri-IoT system performance are relatively untouched areas, as stated in studies [18,22]. The creation of adaptive resource allocation algorithms along with effective task-scheduling methods that are specifically designed for Agricultural IoT settings shows excellent potential for future investigation.

Dealing with difficulties in moving data for IoT devices, especially in situations where cellular networks are overloaded, the study ref.  [23] proposed offloading schemes that depend on prediction and make use of mobility patterns. To solve the problem of resource scheduling in Federated Learning (FL) environments, study ref. [24] introduced the method called DynamicSched, which improves learning accuracy and scheduling efficiency. Paper [25] talked about how Federated Edge Learning (FEEL) was explored using multi-access edge computing (MEC). The authors used the greedy approach to select UEs, which helped with communication overheads and data privacy problems. They presented the Data-Quality Based Scheduling (DQS) algorithm. It takes into account the UE dataset variety and trustworthiness for picking participating devices and optimally distributing bandwidth. However, it does not completely solve the problem of unfair model updates, where some UEs might put more information into the global model than others, causing an imbalance that could impact the fairness and precision of the Federated Learning process. The random sampling method’s stochastic nature adds variability to the training process that might influence convergence properties. Convergence may require additional tuning and monitoring.

Table 1 provides a summary of existing literature on Dynamic Resource Allocation and Task Scheduling in Edge for IoT Applications. Various research papers have suggested new frameworks to boost resource allocation and performance within distributed computing environments. An example of these is the Modified LSTM-based Congestion-Aware Edge Federated Learning (MLSTM-CEFL) model [26]. This framework focuses on congestion-aware timing and combining resources, but might neglect justice in allotting resources. This can cause individuals who receive fewer allocations to experience a decrease in their performance, which could lead to uneven results during model training processes. In the same manner, there is a BiLSTM-based cloud-edge-client Federated Learning system for Mobile Edge Computing (MEC) in 5G networks [27]. It handles conditions that change over time but does not integrate stochastic models or probabilistic methods to represent uncertainties in wireless channel states and data volumes. Pertaining to privacy and security, this TrustFedGRU framework [28] improves Federated Learning (FL) for the Industrial Internet of Things (IIoT) using dynamic update strategies. Yet it exhibits no consideration for non-uniformity in client data distributions (Non-IID) or differing computational abilities, hence indicating potential space for a more flexible strategy. Also, the FedBi-GRU algorithm [29] permits Virtual Network Functions (VNFs) to train locally while sharing model parameters, thereby boosting confidentiality and decreasing computational burden. Nonetheless, it encounters difficulties in adjusting to the changing and non-permanent nature of IoT settings, mainly dealing with abrupt shifts in traffic patterns or resource accessibility. In conclusion, the BILSTM-GRU model for predicting cloud resource use [30] presents enhancements in prediction precision and reduction time when compared to current models. However, it does not tackle fairness concerns regarding resource distribution—a vital matter within cloud settings where several users or applications share identical infrastructure. These models have been used in state-of-the-art research for resource allocation, task scheduling, and performance optimization in distributed computing environments. The chosen models represent a progression in sequence-learning architectures, from basic GRU/LSTM to more complex bidirectional and hybrid models. This allows for a fair comparison of how different architectures impact resource allocation and scheduling performance. These were chosen based on their relevance and common usage in sequence modeling tasks, particularly for time-series data, which is critical in IoT systems. Each model represents a distinct approach to handling sequential dependencies, with varying levels of complexity and performance.

Study [31] proposes an F-DDQN-based framework for energy-efficient resource allocation in D2D-assisted HetNets. It focuses on resource allocation and power control for a limited number of DDPs and CUEs. However, it does not address the scalability challenges arising from the massive number of devices in real-world D2D-assisted 6G networks. Moreover, the static reward function used for optimization does not consider real-time trade-offs among energy efficiency, latency, and throughput. It reduces its practical applicability in various scenarios. Study [32] proposes a high-precision map caching approach for ICVs with FL and Dueling-DQN to optimize vehicle participation and resource allocation. However, it does not consider the impact brought about by non-IID data that always characterizes real-world driving with diverse behaviors, environments, and sensing capabilities. Moreover, while participant selection is optimized per time slice, fairness over time is not guaranteed in the study and may result in the overuse or neglect of some vehicles. In study [33], the DQN-based method is proposed for node selection in FL over a network of data and hardware heterogeneity. Yet it does not exploit any dynamic change in the policy of node selection with respect to changes in the network, in client availability, or even regarding fluctuations in performance across devices. Paper [34] handles offloading decisions in MEC environments using DDQN agents on mobile devices while D3QN agents are on MEC servers; however, they do not cover heterogeneous mobile devices in a Federated Learning setup, considering hardware, processing power, battery life, and network connectivity to preserve the performance.

Various approaches have been proposed for client selection, each with its strengths and limitations. One notable method is the Enhanced Multi-Layer Feedforward Network (EMFFN) [35], which evaluates clients based on metrics such as Received Signal Strength Indicator (RSSI), statistical efficiency, communication efficiency, bandwidth, and energy levels. While EMFFN optimizes FL performance by reducing convergence time and improving accuracy, its reliance on static criteria may hinder adaptability to real-time changes in client performance.

To solve these problems of unfair model updates, privacy issues, and unbalanced resource utilization in edge computing, we suggest using a User-Efficient Federated Learning Algorithm for Resource Allocation in Edge Computing Networks. This can be applied to the Agricultural IoT scenario and it chooses UEs dynamically according to resource availability while also dealing with fairness problems.

## 3. Problem Formulation

This section initiates with an introduction to the system model, encompassing both the offloading and calculation models aided by the IoT. Formulating the Edge-Agri-IoT problem mathematically involves defining the key components, variables, and objectives of the system. We embrace the framework of edge computing, structured around three principal components: edge devices, edge nodes/servers, and clouds. Tasks are assigned to local devices and can be executed on any of these components.

We let $D=\{d_{1},d_{2},\ldots ,d_{n}\}$ represent the set of edge devices deployed in the agricultural environment, $T=\{t_{1},t_{2},\ldots ,t_{m}\}$ denote the set of tasks assigned on the edge devices, and $E=\{e_{1},e_{2},\ldots ,e_{n}\}$ represent the set of edge servers where data can be offloaded for further processing. *C* represents the central cloud server. $x_{ij}\in \left\{0,1\right\}$ represents the binary decision variable indicating whether task $t_{i}$ is assigned to edge device $d_{j}$ ($x_{ij}=1$ if assigned, $x_{ij}=0$ otherwise), and $y_{jk}\in \left\{0,1\right\}$ represents the binary decision variable indicating whether edge device $d_{j}$ offloads data to the edge server $e_{j}$ ($y_{jk}=1$ if offloaded, $y_{jk}=0$ otherwise).

Table 2 describes the variables used in the problem formulation. The objective of our proposed method is to maximize the efficiency and performance of Agricultural IoT applications through the design of an optimized Edge-Agri-IoT architecture. The mathematical interpretation of the objective function is given below:

(1) $$\begin{array}{cc} \mathrm{Maximize}\;Z & =\sum_{i=1}^{m}\sum_{j=1}^{n}\mathrm{Performance}\left(x_{ij}\right)-\lambda \sum_{j=1}^{n}\mathrm{OffloadCost}\left(y_{jk}\right) \end{array}$$

### Constraints

Constraints for the Edge-Agri-IoT problem are as follows:

1. Each task must be assigned to exactly one edge device:
  (2) $$\sum_{j=1}^{n}x_{ij}=1,\;\forall i\in \left\{1,2,\ldots ,m\right\}$$
2. Capacity constraint for each edge device:
  (3) $$\begin{array}{cc} \sum_{i=1}^{m}\mathrm{DataLoad}\left(x_{ij}\right) & \leq \mathrm{Capacity}\left(d_{j}\right),\;\forall j\in \left\{1,2,\ldots ,n\right\} \end{array}$$
3. Offload decision constraint:
  (4) $$\sum_{j=1}^{n}y_{jk}\leq 1,\;\forall k\in \left\{1\right\}$$
4. Data transfer constraint:
  (5) $$\begin{array}{cc} \sum_{i=1}^{m}\mathrm{DataLoad}\left(x_{ij}\right)\cdot x_{ij} & =\sum_{k=1}^{1}\mathrm{DataOffload}\left(y_{jk}\right),\;\forall j\in \left\{1,2,\ldots ,n\right\} \end{array}$$

For resource allocation in edge computing networks within the Agricultural IoT environment, we need to incorporate the impact of user equipment (UE) sampling, unfair model updates, and convergence challenges.

UE Sampling: A subset $K_{g}$ of UEs is randomly selected in each global round.

(6) $$K_{g}\subseteq \mathrm{UEs}$$

(7) $$|K_{g}\left|\leq |\mathrm{UEs}\right|$$

Impact of Stragglers: If some UEs are selected more frequently due to random sampling, they may not contribute equally to the learning process, causing delays in convergence. We can represent this impact by introducing factor $\alpha_{i}$ for each UE, where $\alpha_{i}$ denotes the weight or frequency of selection for UE *i*. Thus, the learning progress of UE *i* can be modified as

(8) $$\Delta x_{i}=\eta \alpha_{i}\frac{\partial f}{\partial x_{i}}$$

Convergence Challenges: The stochastic nature of random sampling introduces variability in the updates, affecting the convergence properties. This variability can be represented by adding random noise to the learning progress updates. Let us denote $\epsilon_{i}$ as the random noise for UE *i*. Then, the learning progress update with convergence challenges can be represented as

(9) $$\Delta x_{i}=\eta \frac{\partial f}{\partial x_{i}}+\epsilon_{i}$$

Overall, the learning progress update for each UE, considering both stragglers and convergence challenges, can be represented as

(10) $$\Delta x_{i}=\eta \left(\alpha_{i}\frac{\partial f}{\partial x_{i}}\right)+\epsilon_{i}$$

These representations capture the impact of stragglers and convergence challenges in the learning process. Adjustments to the learning rate η, as well as additional tuning and monitoring, may be necessary to ensure convergence despite these challenges.

## 4. System Design and Algorithm

**Figure 1:**
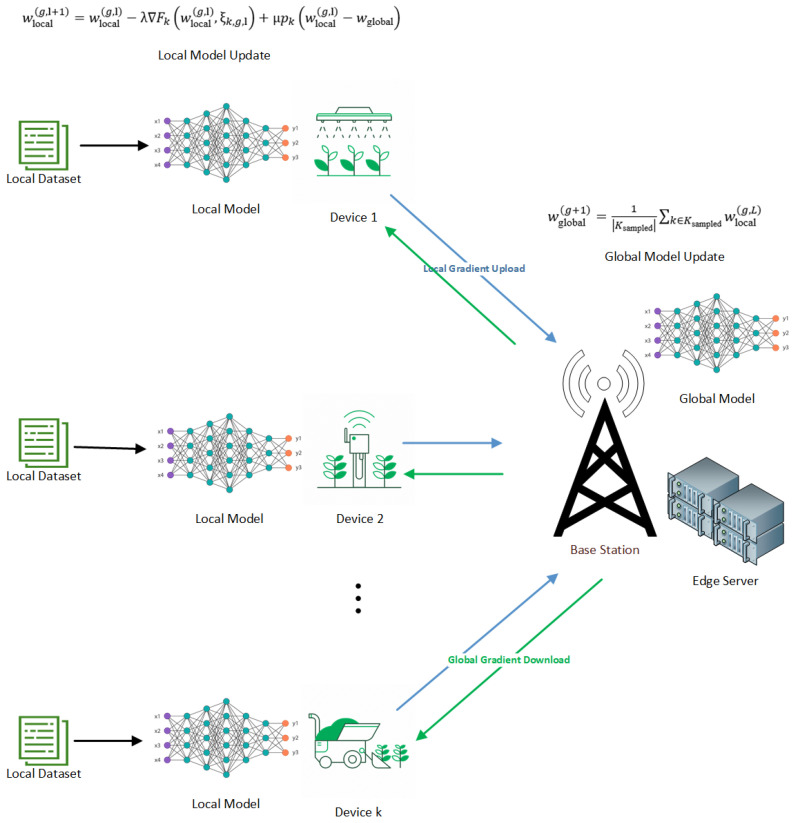
Edge Computing architecture in an agricultural environmental setting.

The system is designed in two parts, namely the local computing model and the edge server computation model, as shown in Figure 1.

### 4.1. Local Computing Model

The objective is to maximize the local computing performance while minimizing the overall energy consumption in a local computing environment. $D=\{d_{1},d_{2},\ldots ,d_{n}\}$ represents the set of local computing edge devices available, $T=\{t_{1},t_{2},\ldots ,t_{m}\}$ denotes the set of computing tasks that need to be processed locally, and $x_{ij}\in \left\{0,1\right\}$ represents the binary decision variable indicating whether task $t_{i}$ is assigned to device $d_{j}$ ($x_{ij}=1$ if assigned, $x_{ij}=0$ otherwise). Table 3 describes the variables used in the local computing model.

#### Objective Function


(11) $$\begin{array}{cc} \; & \mathrm{Maximize}\;\mathrm{Local}\;\mathrm{Computing}\;\mathrm{Performance}:\;Z\\ \; & =\sum_{i=1}^{m}\sum_{j=1}^{n}\mathrm{PerformanceMetric}\left(x_{ij}\right)-\lambda \sum_{j=1}^{n}\mathrm{EnergyConsumption}\left(d_{j}\right) \end{array}$$

### 4.2. Edge Server Computation Model

#### 4.2.1. Signal-to-Noise Ratio (SNR)

The signal-to-noise ratio between the edge device and the edge server is given by

(12) $$\gamma_{e_{j},t}=\frac{P_{u}\cdot h_{e_{j},t}}{\sum_{t^{\prime }\in T\setminus \left\{t\right\}}P_{u}\cdot n_{t}\cdot h_{n,t^{\prime },t}+\sigma^{2}}$$

This equation calculates the SNR by dividing the signal power $P_{u}\cdot h_{e_{j},t}$ by the sum of noise and interference powers. The interference power is represented by the sum over all tasks $t^{\prime }$ in set *T* (excluding the current task *t*) of $P_{u}\cdot n_{t}\cdot h_{n,t^{\prime },t}$, which captures the interference caused by other tasks on the channel. This sum is then added to the noise power $\sigma^{2}$.

This equation provides a measure of the quality of the communication channel between the end user and the edge server for a specific task *t*, considering both the signal power and the effects of noise and interference. Higher SNR values indicate better signal quality relative to noise and interference.

#### 4.2.2. Objective Function

The objective is to maximize the edge server computation performance while minimizing the overall energy consumption:

(13) $$\begin{array}{c} Z=\sum_{i=1}^{m}\sum_{j=1}^{n}\mathrm{PerformanceMetric}\left(x_{ij}\right)-\lambda \sum_{j=1}^{n}\mathrm{EnergyConsumption}\left(e_{j}\right) \end{array}$$

### 4.3. Offload to Edge Servers

The data transfer rate $R_{e_{j},t}$ at which the end user offloads the computational task to the edge server is given by

(14) $$R_{e_{j},t}=B_{e_{j},t}\cdot \log_{2}\left(1+\gamma_{e_{j},t}\right)$$

#### 4.3.1. Data Transfer Delay

The data transfer delay $D_{\mathrm{transfer}}$ for offloading computational tasks from an end user to an edge server is

(15) $$D_{\mathrm{transfer}}=\frac{D_{\mathrm{data}}}{B_{e_{j},t}\cdot \log_{2}\left(1+\gamma_{e_{j},t}\right)}$$

#### 4.3.2. Computational Delay

The computational delay $D_{\mathrm{compute}}$ in offloading a computational task to an edge server involves the time it takes for the edge server to process the task:

(16) $$D_{\mathrm{compute}}=\frac{C_{t}}{F_{e_{j}}}$$

#### 4.3.3. Transmission Rate

The transmission rate $R_{\mathrm{transmission}}$ of the calculated result data from the edge server back to the end devices is given by

(17) $$R_{\mathrm{transmission}}=B_{e_{j}}\cdot \log_{2}\left(1+\gamma_{e_{j},t}\right)$$

#### 4.3.4. Return Delay

The return delay $D_{\mathrm{return}}$ of the calculated result data from the edge server to the end user is given by

(18) $$D_{\mathrm{return}}=\frac{D_{\mathrm{result}}}{R_{\mathrm{transmission}}}$$

#### 4.3.5. Energy Consumption

The energy consumption of edge server transmission during the execution of a computational task is

(19) $$E_{\mathrm{transmission}}=P_{i}\cdot T_{n_{i},j}+P_{e}\cdot T_{n_{j},i}$$

#### 4.3.6. Computational Energy Consumption

The computational energy consumption $E_{\mathrm{compute}}$ during the execution of a computational task by an edge server is given by

(20) $$E_{\mathrm{compute}}=P_{\mathrm{compute}}\cdot C_{t}$$

#### 4.3.7. Total Energy Consumption

The total energy consumption $E_{\mathrm{Total}}$ during the execution of a computational task by an edge server is

(21) $$E_{\mathrm{Total}}=E_{\mathrm{compute}}+E_{\mathrm{transmission}}$$

### 4.4. Objective in Federated Learning

The objective in Federated Learning involves collaborative training of a global model across distributed user devices (UEs), all without central aggregation of sensitive data. Nevertheless, an issue emerges: the potential for unfairness in model updates—certain UEs may contribute disproportionately to the training process—and this could result in biased or suboptimal global models. In response to this challenge, a solution is proposed: an algorithm for Federated Learning that dynamically determines the subset of user equipment (UEs) participating in every global iteration, guided by a Reinforcement Learning (RL) agent. The aim is to minimize the global objective function and ensure fairness in model updates, focusing on this particular objective.

The global objective function in this context aims to minimize the overall loss incurred by the system while addressing the issue of unfair model updates. Mathematically, the global objective function can be defined as follows: We let $L_{k}\left(w_{k}\right)$ represent the local loss function for UE *k* with model parameters $w_{k}$ and dataset $D_{k}$. Then, the global objective function can be defined as the average of the local loss functions over all selected UEs, with a regularization term to ensure fairness in sampling:

(22) $$\min_{w}\frac{1}{|K_{\mathrm{sampled}}|}\sum_{k\in K_{\mathrm{sampled}}}L_{k}\left(w_{k}\right)+\lambda \sum_{k=1}^{K_{\mathrm{tot}}}p_{k}\cdot \left|\right|w_{k}-w_{\mathrm{global}}{\left|\right|}^{2}$$

Table 4 describes the variables used in the Objective in Federated Learning.

This objective function seeks to minimize the average local loss across the selected UEs while also penalizing large deviations of local models from the global model, thus promoting fairness in updates. The regularization term encourages the local models to stay close to the global model, which helps in achieving fairness in contributions across different UEs.

The objective is to minimize the global objective function subject to the given constraints:

(23) $$\begin{array}{cc} \mathrm{Minimize}:\;J(w_{\mathrm{global}}) & =\frac{1}{|K_{\mathrm{sampled}}|}\sum_{k\in K_{\mathrm{sampled}}}L_{k}\left(w_{k}\right)+\lambda \sum_{k=1}^{K_{\mathrm{tot}}}p_{k}\cdot \left|\right|w_{k}-w_{\mathrm{global}}{\left|\right|}^{2} \end{array}$$

Subject to

1. $w_{k}=w_{\mathrm{global}}$ for all *k* in $K_{\mathrm{sampled}}$ after local training;
2. $w_{\mathrm{global}}=\frac{1}{|K_{\mathrm{sampled}}|}\sum_{k\in K_{\mathrm{sampled}}}w_{k}$ after global model aggregation.

The constraints ensure that after local training, the local models are synchronized with the global model, and after aggregation, the global model is updated based on the contributions from the selected UEs.

This optimization problem seeks to find the optimal global model parameters $w_{\mathrm{global}}$ that minimize the average local loss across the selected subset of UEs while encouraging fairness in updates by penalizing large deviations of local models from the global model. The constraints ensure that after local training, the local models are synchronized with the global model, and after aggregation, the global model is updated based on the contributions from the selected UEs.

### 4.5. Proposed Algorithm

#### 4.5.1. Task Offloading Algorithm

Emphasizing the significance of Borg cells in large-scale cluster management in [36,37], our project leverages Google Cluster Traces, a dataset replete with detailed information. This indispensable resource includes CPU usage histograms, allocation set details, and job parent data that span several days of workload on a single Borg cell. Given its massive size, approximately 2.4 TiB compressed, we turn to Google BigQuery for sophisticated analysis. For the analysis, we use the instance usage table of the dataset.

##### Bidirectional LSTM Layer

**Figure 2:**
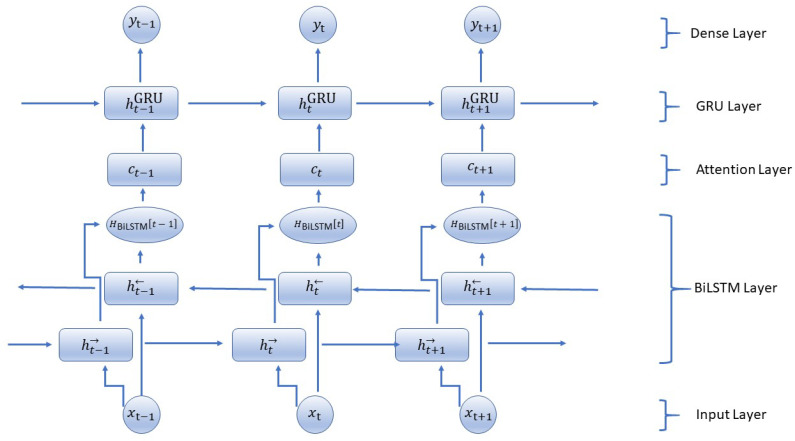
Hybrid model architecture.

The Figure 2 shows the architectural details of the proposed BiLSTM-GRU model. The proposed BiLSTM-GRU model (Algorithm 1) starts by initializing the weights and biases for the LSTM, GRU, and Dense layers, as well as the initial hidden and cell states for both the forward and backward LSTM layers and the initial hidden state for the GRU layer. The input sequence is now implemented into the Bidirectional LSTM layer. For every time step *t*, the forward LSTM calculates the forward hidden state and cell state by using the input at *t* along with the previous hidden state. The backward LSTM also works similarly, but it processes sequences in reverse order to compute the backward hidden state and the cell state. The output of this layer at each time step is formed by combining both forward and backward hidden states. BiLSTM is used for capturing dependencies from past and future, GRU for effective memory management, and an attention mechanism to emphasize necessary time steps making the model capable of providing high accuracy in predicting time-series tasks. The combination of these architectures can be scaled up to large datasets efficiently, particularly with real-time IoT environments. By concentrating on vital data points only through an attention mechanism, this model deals with vast input sequences effectively; at the same time, BiLSTM and GRU layers keep their potential for forming complex temporal patterns throughout larger sequences intact. In uses such as intelligent farming, health surveillance, and industry IoT, where real-time predictions are critical, this mixed structure supports swift choices based on the past and forthcoming environment.
**Algorithm 1** Proposed BiLSTM-GRU with Attention Mechanism**Input:** Sequential input data $X=\{x_{1},x_{2},\ldots ,x_{T}\}$, where each $x_{t}$ is a feature vector at time *t***Output:** Predicted output sequence $\hat{y}=\left\{y_{1},y_{2},\ldots ,y_{T}\right\}$ after applying the BiLSTM-GRU model with attention 1:**Initialization:** Initialize model weights and biases: $W_{\mathrm{lstm}}$, $U_{\mathrm{lstm}}$, $b_{\mathrm{lstm}}$, $W_{a}$, $b_{a}$, $W_{\mathrm{gru}}$, $U_{\mathrm{gru}}$, $b_{\mathrm{gru}}$, $W^{\mathrm{dense}}$, $b^{\mathrm{dense}}$ 2:Initialize hidden states and cell states: $h_{t}^{\to }$, $h_{t}^{\leftarrow }$, $c_{t}^{\to }$, $c_{t}^{\leftarrow }$, $h_{t-1}^{\mathrm{GRU}}$ 3:**Bidirectional LSTM Layer:** 4:**for** (t=0,1,…,T−1) **do** 5: Forward LSTM step: Compute forward hidden state $h_{t}^{\to }$ and cell state $c_{t}^{\to }$ 6: Backward LSTM step: Compute backward hidden state $h_{t}^{\leftarrow }$ and cell state $c_{t}^{\leftarrow }$ 7: Concatenate forward and backward hidden states: $H_{\mathrm{BiLSTM}}\left[t\right]$ 8:**end for** 9:**Attention Mechanism:**10:**for** (t=0,1,…,T−1) **do**11: Compute attention weights for each time step: $e_{t}=\tanh\left(W_{a}H_{\mathrm{BiLSTM}}\left[t\right]+b_{a}\right)$12: Normalize attention weights: $\alpha_{t}=\mathrm{softmax}\left(e_{t}\right)$13: Compute context vector as a weighted sum of hidden states: $c_{t}=\sum_{i=0}^{T-1}\alpha_{i}\cdot H_{\mathrm{BiLSTM}}{\left[t\right]}_{i}$14: Concatenate context vector with current BiLSTM hidden state: $h_{\mathrm{att}\_t}=\left[c_{t};H_{\mathrm{BiLSTM}}\left[t\right]\right]$15:**end for**16:**Gated Recurrent Unit (GRU) Layer:**17:**for** (t=0,1,…,T−1) **do**18: GRU forward step: Compute update gate $z_{t}$, reset gate $r_{t}$, candidate hidden state ${\tilde{h}}_{t}$19: Update hidden state: $h_{t}^{\mathrm{GRU}}$ using $h_{\mathrm{att}\_t}$ as input20:**end for**21:**Dense Layer:**22:Perform linear transformation: $z=h_{t}^{\mathrm{GRU}}\cdot W^{\mathrm{dense}}+b^{\mathrm{dense}}$23:Apply activation function: y=softmax(z)24:**Training, Evaluation, and Prediction:**25:Train model using backpropagation and optimization algorithm26:Evaluate model performance using appropriate metrics27:Make predictions on new sequential data

For a given input sequence $X=\left(x_{1},x_{2},\ldots ,x_{T}\right)$ of length *T*, a BiLSTM layer processes it in both forward (→) and backward (←) directions. The forward hidden states $h_{t}^{\to }$ and backward hidden states $h_{t}^{\leftarrow }$ at each time step *t* are computed as follows:

Forward LSTM:

(24) $$i_{t}^{\to }=\sigma \left(W_{ii}^{\to }x_{t}+b_{ii}^{\to }+W_{hi}^{\to }h_{t-1}^{\to }+b_{hi}^{\to }\right)$$

(25) $$f_{t}^{\to }=\sigma \left(W_{if}^{\to }x_{t}+b_{if}^{\to }+W_{hf}^{\to }h_{t-1}^{\to }+b_{hf}^{\to }\right)$$

(26) $$g_{t}^{\to }=\tanh\left(W_{ig}^{\to }x_{t}+b_{ig}^{\to }+W_{hg}^{\to }h_{t-1}^{\to }+b_{hg}^{\to }\right)$$

(27) $$o_{t}^{\to }=\sigma \left(W_{io}^{\to }x_{t}+b_{io}^{\to }+W_{ho}^{\to }h_{t-1}^{\to }+b_{ho}^{\to }\right)$$

(28) $$c_{t}^{\to }=f_{t}^{\to }⊙c_{t-1}^{\to }+i_{t}^{\to }⊙g_{t}^{\to }$$

(29) $$h_{t}^{\to }=o_{t}^{\to }⊙\tanh\left(c_{t}^{\to }\right)$$

Backward LSTM:

(30) $$i_{t}^{\leftarrow }=\sigma \left(W_{ii}^{\leftarrow }x_{t}+b_{ii}^{\leftarrow }+W_{hi}^{\leftarrow }h_{t+1}^{\leftarrow }+b_{hi}^{\leftarrow }\right)$$

(31) $$f_{t}^{\leftarrow }=\sigma \left(W_{if}^{\leftarrow }x_{t}+b_{if}^{\leftarrow }+W_{hf}^{\leftarrow }h_{t+1}^{\leftarrow }+b_{hf}^{\leftarrow }\right)$$

(32) $$g_{t}^{\leftarrow }=\tanh\left(W_{ig}^{\leftarrow }x_{t}+b_{ig}^{\leftarrow }+W_{hg}^{\leftarrow }h_{t+1}^{\leftarrow }+b_{hg}^{\leftarrow }\right)$$

(33) $$o_{t}^{\leftarrow }=\sigma \left(W_{io}^{\leftarrow }x_{t}+b_{io}^{\leftarrow }+W_{ho}^{\leftarrow }h_{t+1}^{\leftarrow }+b_{ho}^{\leftarrow }\right)$$

(34) $$c_{t}^{\leftarrow }=f_{t}^{\leftarrow }⊙c_{t+1}^{\leftarrow }+i_{t}^{\leftarrow }⊙g_{t}^{\leftarrow }$$

(35) $$h_{t}^{\leftarrow }=o_{t}^{\leftarrow }⊙\tanh\left(c_{t}^{\leftarrow }\right)$$

The provided equations delineate the forward and backward LSTM units’ calculations essential for processing such data. First, the input, forget, output, and input gates for the forward and backward LSTMs ($i_{t}^{\to }$, $f_{t}^{\to }$, $o_{t}^{\to }$, $g_{t}^{\to }$; $i_{t}^{\leftarrow }$, $f_{t}^{\leftarrow }$, $o_{t}^{\leftarrow }$, $g_{t}^{\leftarrow }$) are delineated. These gates regulate the flow of information, enabling the model to selectively retain or discard information at each time step. Additionally, the equations illustrate how the cell states ($c_{t}^{\to }=f_{t}^{\to }⊙c_{t-1}^{\to }+i_{t}^{\to }⊙g_{t}^{\to }$, $c_{t}^{\leftarrow }=f_{t}^{\leftarrow }⊙c_{t+1}^{\leftarrow }+i_{t}^{\leftarrow }⊙g_{t}^{\leftarrow }$) are updated. The new cell states are computed by combining the previous cell states with selectively weighted information using the input and forget gates. Lastly, the equations detail the computation of hidden states ($h_{t}^{\to }=o_{t}^{\to }⊙\tanh\left(c_{t}^{\to }\right)$, $h_{t}^{\leftarrow }=o_{t}^{\leftarrow }⊙\tanh\left(c_{t}^{\leftarrow }\right)$), which encapsulate the model’s learned representation of the input sequence. These hidden states are influenced by the cell states and are modulated by the output gates, enabling the model to capture and retain relevant information for prediction tasks. Together, these computations empower the BiLSTM to effectively process sequential data, learning intricate dependencies both forward and backward through time.

##### Attention Mechanism Integration

The novel aspect of adding the attention mechanism to the BiLSTM-GRU model lies in its ability to selectively focus on the most relevant time steps in the input sequence. For each time step, an attention score is computed using a learned weight matrix $W_{a}$ and the hidden states from the BiLSTM layer. This score reflects how much attention the model should pay to a particular time step.

Step 1: Compute Attention Weights. First, we compute the attention weights for the hidden state output by the BiLSTM. The attention weights help the model to decide which parts of the sequence are more important at each time step.

For each hidden state $H_{\mathrm{BiLSTM}}\left[t\right]$ of the BiLSTM, we compute the attention score $e_{t}$:

(36) $$e_{t}=\tanh\left(W_{a}H_{\mathrm{BiLSTM}}\left[t\right]+b_{a}\right)$$

where $W_{a}$ and $b_{a}$ are learnable parameters.

Next, we compute the attention weights $\alpha_{t}$ using a softmax function:

(37) $$\alpha_{t}=\frac{\exp(e_{t})}{\sum_{t^{\prime }}\exp\left(e_{t^{\prime }}\right)}$$

Step 2: Compute Context Vector. Using the attention weights, we compute the context vector $c_{t}$, which is a weighted sum of the hidden states:

(38) $$c_{t}=\sum_{t^{\prime }}\alpha_{t^{\prime }}H_{\mathrm{BiLSTM}}\left[t^{\prime }\right]$$

Step 3: Combine Context Vector with GRU. We integrate the context vector into the GRU computations. Instead of directly feeding the hidden states from the BiLSTM to the GRU, we combine them with the context vector.

We modify the GRU equations as follows:

Update Gate ($z_{t}$):

(39) $$z_{t}=\sigma \left(W_{z}\cdot \left[h_{t-1}^{\mathrm{GRU}},c_{t},H_{\mathrm{BiLSTM}}\left[t\right]\right]+b_{z}\right)$$

Reset Gate ($r_{t}$):

(40) $$r_{t}=\sigma \left(W_{r}\cdot \left[h_{t-1}^{\mathrm{GRU}},c_{t},H_{\mathrm{BiLSTM}}\left[t\right]\right]+b_{r}\right)$$

Candidate Hidden State ($\tilde{h_{t}}$):

(41) $$\tilde{h_{t}}=\tanh\left(W_{h}\cdot \left[r_{t}⊙h_{t-1}^{\mathrm{GRU}},c_{t},H_{\mathrm{BiLSTM}}\left[t\right]\right]+b_{h}\right)$$

Hidden State Update ($h_{t}^{\mathrm{GRU}}$):

(42) $$h_{t}^{\mathrm{GRU}}=\left(1-z_{t}\right)⊙h_{t-1}^{\mathrm{GRU}}+z_{t}⊙\tilde{h_{t}}$$

##### Dense Layer

The outcome of the last hidden state, which comes from the GRU layer at the final time step, flows into a Dense layer. Here, there is a linear change, and then the use of the softmax activation function to provide a result or output that represents the model’s predictions. By performing backpropagation and using an optimization algorithm, we train the model. After this training process is finished, we evaluate it with suitable metrics before finally making predictions on fresh sequential data. The model is improved by using layers of Bidirectional LSTM and GRU together, along with the attention mechanism. This lets the model understand relationships in the past as well as future elements of the sequence, making it better at prediction.

(43) $$z=h_{t}^{\mathrm{GRU}}\cdot W^{\mathrm{dense}}+b^{\mathrm{dense}}$$

(44) y=softmax(z)

The task offloading Algorithm 2 starts by setting a counter to zero. It uses a model that has already been trained for predicting the resource needs of tasks as time goes on, which we represent with the workload matrix W(t). For each edge server *i*, it calculates forecasted resource requirements $P_{i}$ by using the prediction model on present data $D_{i}$.
**Algorithm 2** Task Offloading Algorithm**Input:** Workload matrix W(t) representing the resource requirements of tasks over time and Edge server information matrix EdgeServerInfo**Output:** Predicted data, Energy consumption matrix, Delay matrix, Cost matrix 1:Initialize Counter=0 2:Load a pre-trained model for predicting task resource requirements 3:**for** each edge server *i* **do** 4: Calculate $P_{i}=\mathrm{Predict}\left(D_{i}\right)$ 5:**end for** 6:**for** each edge server *i* **do** 7: Calculate $F_{i}=\mathrm{Combine}\left(P_{i},E_{i},\Delta_{i},C_{i}\right)$ 8:**end for** 9:Sort edge servers based on the decision factor: $\mathrm{SortedServers}=\mathrm{Sort}(F_{i})$10:**for** each service **do**11: **if** $F_{i}<\mathrm{Threshold}$ **then**12:  Process the service locally13: **else**14:  Migrate the service to the selected edge server15: **end if**16:**end for**17:**for** each edge server *i* in SortedServers **do**18: **if** the edge server has enough capacity to host the current service and the service is not already hosted **then**19:  Provision(i)20:  Increment Counter21:  Exit the loop to ensure the service is migrated only once22: **end if**23:**end for**24:**if** Counter=N **then**25: Halt the process26:**end if**

Then, we calculate a decision factor $F_{i}$ for every edge server. It is obtained by combining predicted resource requirements $P_{i}$, energy consumption $E_{i}$, delay $\Delta_{i}$, and cost $C_{i}$. After that, the list of edge servers is sorted. The result becomes a sorted list SortedServers.

The algorithm continues by checking each service. If the decision factor $F_{i}$ is less than a specific limit, then this service is handled locally on the edge server. Otherwise, it needs to move over and be processed at the chosen edge server from the sorted list.

For every edge server in the list that has been sorted, we see if it can hold the service and make sure this service is not already hosted on that server. If these conditions are fulfilled, we move the service to this edge server, increase our counter by one, and stop the loop so that migration only happens once for each service.

Lastly, if the counter equals the total number of services *N*, then it stops. This shows that all services have been allocated well on edge servers. This algorithm manages task offloading in a good way. It uses prediction models and thinks about many things like energy use, delay, cost, etc., to make sure resources are put into edge computing places as much as possible.

The process of the hybrid migration algorithm is supported by a discrete-event simulator called EdgeSimPy. This tool is purposely designed for modeling edge computing scenarios, making it easier to create situations that involve operations on edge servers and allowing resource management as well as analysis of system behavior [38].

The script makes use of the Pandas library to load a JSON file, which contains comprehensive data regarding edge servers. It then arranges this information into an array of dictionaries. Each dictionary represents a single server with distinct attributes such as ‘cpu_demand’ and ‘memory_demand’. Feature engineering is carried out for predictive enhancement using scikit-learn classes. The script employs TensorFlow’s Keras API to load a pre-trained Bidirectional LSTM model to predict CPU and memory usage for each edge server. It simulates decision-making logic to determine if a service should be processed locally or migrated based on predicted resource usage, optimizing edge server categorization and decision-making. If there is a need for migration, the service is provided to a server that has capacity, and migration statistics are updated.

An object made with EdgeSimPy simulates edge computing situations using the dataset. The simulation continues until a stopping point is reached, which checks whether every service has been successfully provided.

The ’hybrid_offloading_algorithm’ function manages offloading and migration decisions, utilizing predictions from a pre-trained hybrid model of BiLSTM and GRU. The script employs EdgeSimPy’s helper methods to iterate over all edge servers and services, implementing the hybrid migration logic and maintaining a record of migration counts.

### 4.6. Proposed Federated Learning Algorithm with Dynamic UEs Selection

The start of this process is marked by setting up the global model weights ($w_{\mathrm{global}}$) and regularization parameters (λ), which are uniform for all user equipment (UE). An agent for D4PG starts next, having been given appropriate observation and action spaces to help it make decisions. In every overall iteration (*g*), the agent chooses a subset ($K_{\mathrm{sampled}}$) from all available UEs ($K_{g}$), and this selection changes dynamically using policies learned to optimize the system’s general performance.

Next, when the subset of UEs is chosen, the present global model ($w_{\mathrm{global}}$) is sent to these sampled UEs. Each UE (*k*) in this set starts its local model ($w_{\mathrm{local}}$) by using weights from the global model it received earlier. Then, UEs proceed with local training for *L* rounds. In every iteration (*l*), we pick one random data point ($\xi_{k,g,l}$) from the local dataset ($D_{k}$) of the UE and update its local model with the gradient descent method. The formula for updating includes a regularization term ($\mu p_{k}$) that helps in balancing how much the local model deviates from the global one:

(45) $$\begin{array}{cc} w_{{\left(g,l+1\right)}_{\mathrm{local}}} & =w_{{\left(g,l\right)}_{\mathrm{local}}}-\lambda \nabla F_{k}\left(w_{{\left(g,l\right)}_{\mathrm{local}}},\xi_{k,g,l}\right)+\mu p_{k}\left(w_{{\left(g,l\right)}_{\mathrm{local}}}-w_{\mathrm{global}}\right) \end{array}$$

When the local training is finished, every UE sends its new local model to the base station. The base station combines all these local models into a fresh global model ($w_{{\left(g+1\right)}_{\mathrm{global}}}$) by obtaining an average of weights from chosen UEs:

(46) $$w_{\mathrm{global}}^{\left(g+1\right)}=\frac{1}{\left|K_{\mathrm{sampled}}\right|}\sum_{k\in K_{\mathrm{sampled}}}w_{\mathrm{local}}^{\left(g,L\right)}+N\left(0,\sigma^{2}I\right)$$

Updates to the model based on feedback about fairness and effectiveness are utilized for adjusting the policies of the Reinforcement Learning agent. Therefore, this helps in enhancing its ability to choose optimal UEs during subsequent iterations. The iterative process carries on for a fixed number of global iterations (*G*), gradually improving the global model via collaborative learning and adaptive sampling techniques.

#### 4.6.1. Objective

The global objective function can be minimized by updating the global model parameters. This is important because it ensures that unfairness in model updates, where some UEs contribute more often than others, is taken care of.

Traditional Federated Learning algorithms often select a static or random subset of UEs for local training in each iteration [39,40,41]. In contrast, this Algorithm 3 leverages a D4PG agent to dynamically learn policies for selecting an optimal subset of UEs, Algorithm 4. The selection is based on observed states (UE availability, network conditions, resource constraints) and actions that maximize the overall training efficiency and fairness. This policy-driven approach to selection is a key differentiator from standard random or fixed strategies.
**Algorithm 3** Federated Learning Algorithm with Dynamic UE Selection using D4PG**Input:** Global model parameters $w_{\mathrm{global}}$, UE dataset $D_{k}$ for each UE *k*, hyperparameter λ for all UEs, maximum number of global iterations *G*, local training iterations *L*.**Output:** Final global model parameters $w_{\mathrm{global}}^{\left(G\right)}$, trained D4PG agent, performance metrics. 1:**Initialization:** Initialize global model parameters $w_{\mathrm{global}}$. Initialize the hyperparameter λ for all UEs. 2:**D4PG Agent Initialization:** Initialize the D4PG agent with appropriate observation and action spaces, neural networks for policy and value functions, and experience replay buffer. 3:**Dynamic Sampling:** At each global iteration *g*: 4: D4PG agent dynamically selects a subset $K_{\mathrm{sampled}}$ of $K_{g}$ UEs based on learned policies 5:**Global Model Broadcast:** Broadcast the global model parameters $w_{\mathrm{global}}$ to all UEs in $K_{\mathrm{sampled}}$. 6:**Local Training:** Each UE $k\in K_{\mathrm{sampled}}$ performs local training: 7: Initialize local model $w_{\mathrm{local}}$ with $w_{\mathrm{global}}$. 8:**for** (l=0,1,…,L−1) **do** 9:   Randomly select a data point $\xi_{k,g,l}$ from local dataset $D_{k}$.10:  Update local model: As shown in Equation (45)11:**end for**12:**Send Local Models:** Each UE sends its updated local model $w_{\mathrm{local}}^{\left(g,L\right)}$ to the base station.13:**Global Model Aggregation:** The base station aggregates the local models to update the global model: As shown in Equation (46)14:**D4PG Agent Update:** Update the D4PG agent using the reward based on the performance and fairness of the aggregated model, storing experiences in the replay buffer, and training the neural networks.15:**Repeat:** Repeat the process for *G* global iterations.

**Algorithm 4** Dynamic Sampling with D4PG**Input:** Environment, replay buffer *D*, Q-network parameters $\theta_{\mathrm{online}}$ and $\theta_{\mathrm{target}}$, exploration rate ϵ, learning rate α, discount factor γ, target network update frequency *C*, target network update rate τ**Output:** Optimal action sequence for task offloading and resource allocation
 1:Initialize $\theta_{\mathrm{online}}$ and $\theta_{\mathrm{target}}$ 2:**for** each global iteration *g* **do** 3:  Observe current state $s_{g}$ 4:  Select action $a_{g}$: 5:  **if** random(0,1)<ϵ **then** 6:   Choose a random subset of UEs as $a_{g}$ 7:  **else** 8:   Choose $a_{g}=\arg\max_{a}Q\left(s_{g},a;\theta_{\mathrm{online}}\right)$ 9:  **end if**10: Sample subset $K_{\mathrm{sampled}}$ from $a_{g}$11: Record $\left(s_{g},a_{g}\right)$ in *D*12: Conduct local training and obtain reward *R*13: Sample mini-batch $\left(s,a,r,s^{\prime }\right)$ from *D*14: Set target:15: **if** $s^{\prime }$ is terminal **then**16:  target=r17: **else**18:  $target=r+\gamma E[Q\left(s^{\prime },a^{\prime };\theta_{\mathrm{target}}\right)]$19: **end if**20: Update $\theta_{\mathrm{online}}$ by minimizing the loss:$$L={\left(Q(s,a;\theta_{\mathrm{online}})-target\right)}^{2}$$21: Every *C* steps, update $\theta_{\mathrm{target}}$ by:$$\theta_{\mathrm{target}}\leftarrow \tau \theta_{\mathrm{online}}+\left(1-\tau \right)\theta_{\mathrm{target}}$$22:
**end for**



#### 4.6.2. D4PG Agent Initialization

- State Space: $S_{s}=\left\{s_{1},s_{2},\ldots ,s_{|S_{s}|}\right\}$, where $|S_{s}|$ is the size of the state space.
- Action Space: $A_{a}=\left\{a_{1},a_{2},\ldots ,a_{|A_{a}|}\right\}$, where $|A_{a}|$ is the size of the action space.
- Q-function Approximator: Q(s,a;θ) with neural network parameters θ.
  − The Q-function approximator estimates the expected future reward when taking action *a* in state *s*.
  − θ represents the parameters of the neural network.
- Experience Replay Buffer: $D=\{\left(s_{1},a_{1},r_{1},s_{1}^{\prime }\right),\left(s_{2},a_{2},r_{2},s_{2}^{\prime }\right),\ldots ,\left(s_{N},a_{N},r_{N},s_{N}^{\prime }\right)\}$, where *N* is the size of the replay buffer, $r_{i}$ is the reward, and $s_{i}^{\prime }$ is the next state.

#### 4.6.3. Dynamic Sampling Mechanism

The dynamic sampling of action subsets from the action space is a key novelty in this algorithm.

Action Selection in D4PG

In standard D4PG, the action is selected by maximizing the Q-function:

(47) $$a_{g}=\arg\max_{a}Q\left(s_{g},a;\theta_{\mathrm{online}}\right)$$

This deterministic action selection works well for small action spaces.

Dynamic Sampling for Subset of Actions Instead of selecting a single action, we sample a subset $K_{\mathrm{sampled}}\subseteq A$ from the total action space A.

- Exploration vs Exploitation (Epsilon-Greedy Policy): With probability ϵ, a random subset of actions is selected, and with probability 1−ϵ, we maximize the Q-value:
  (48) $$a_{g}=\left\{\begin{array}{cc} \mathrm{Random}\;\mathrm{subset}\;\mathrm{of}\;A, & \mathrm{with}\;\mathrm{probability}\;\epsilon \\ \arg\max_{a\in A}Q\left(s_{g},a;\theta_{\mathrm{online}}\right), & \mathrm{with}\;\mathrm{probability}\;1-\epsilon \end{array}\right.$$
- Dynamic Subset Sampling: After selecting an action $a_{g}$, we sample a subset $K_{\mathrm{sampled}}$ from A:
  (49) $$K_{\mathrm{sampled}}∼P\left(K|s_{g},a_{g}\right)$$
  where $P(K|s_{g},a_{g})$ is a probability distribution over subsets of the action space.

Initially, the online and target Q-network parameters are set up. During each global iteration, the agent observes the current state and selects an action based on an exploration–exploitation strategy: with probability ϵ, it randomly chooses an action, and otherwise it selects the action that maximizes the Q-value from the online Q-network. The agent then samples a subset of actions and records the state-action pair in the replay buffer for training. After taking the action, the agent receives a reward and samples a mini-batch of experiences from the buffer to update the Q-network. The target Q-value is computed using the reward and the expected Q-value of the next state, and the online Q-network is updated by minimizing the loss between the predicted and target Q-values. Every *C* step, the target network is updated by slowly blending the online Q-network’s weights. This process continues iteratively, allowing the agent to learn an optimal policy for task offloading and resource allocation, leveraging both exploration and exploitation while maintaining stable learning with the target network.

#### 4.6.4. Experience Replay Mechanism

Experience replay helps sample past experiences from a replay buffer *D* to improve sample efficiency.

Standard Experience Replay In D4PG, a mini-batch B of past experiences is sampled:

(50) $$L\left(\theta_{\mathrm{online}}\right)=E_{(s,a,r,s^{\prime })∼B}\left[{\left(Q\left(s,a;\theta_{\mathrm{online}}\right)-\mathrm{target}\right)}^{2}\right]$$

where the target is defined as

(51) $$\mathrm{target}=\left\{\begin{array}{cc} r, & \mathrm{if}\;s^{\prime }\;\mathrm{is}\;\mathrm{terminal}\\ r+\gamma E[Q\left(s^{\prime },a^{\prime };\theta_{\mathrm{target}}\right)], & \mathrm{otherwise} \end{array}\right.$$

Experience Replay with Dynamic Sampling In this algorithm, the experience tuples now contain action subsets:

(52) $$D=\{\left(s_{g},K_{\mathrm{sampled}},r_{g},s_{g}^{\prime }\right)\}$$

The mini-batch is sampled from the replay buffer, and the Q-learning loss is modified:

(53) $$L\left(\theta_{\mathrm{online}}\right)=E_{(s,K_{\mathrm{sampled}},r,s^{\prime })∼B}\left[{\left(Q(s,K_{\mathrm{sampled}};\theta_{\mathrm{online}})-\mathrm{target}\right)}^{2}\right]$$

With the target remaining the same:

(54) $$\mathrm{target}=r+\gamma E\left[\max_{a^{\prime }}Q\left(s^{\prime },a^{\prime };\theta_{\mathrm{target}}\right)\right]$$

- Dynamic Subset Selection: The algorithm selects a subset of actions $K_{\mathrm{sampled}}$, allowing for flexibility in handling large or combinatorial action spaces, particularly in distributed or multi-agent environments.
- Sampling Distribution: The probability distribution $P(K|s_{g},a_{g})$ introduces a dynamic and state-dependent sampling mechanism, improving exploration efficiency.
- Experience Replay with Action Subsets: The replay buffer stores subsets of actions rather than single actions, enabling richer learning from diverse experiences, which is especially useful in multi-agent scenarios.

Table 5 describes the architecture details for the BiLSTM-GRU Model with attention mechanism.

## 5. Experimental Results

The experiment was conducted using a system equipped with Intel® Core™ Ultra 7 155H. The system was paired with an NVIDIA® GeForce RTX™ 4070 Laptop GPU, which has 8 GB of GDDR6 VRAM. Additionally, the system included 32 GB of LPDDR5X-7467MHz RAM. For comparison with state-of-the-art methods, the implementation was carried out using TensorFlow 2.14.1. The proposed methodology incorporates a BiLSTM-GRU hybrid model for task scheduling, built using Keras, along with Federated Reinforcement Learning for dynamic resource allocation. The Flower simulation framework [42] 1.6.0 and EdgeSimPy [38] v1.1.0 was used for conducting the simulations. EdgeSimPy provides a modular, scalable environment that supports real-time monitoring, making it an ideal fit for IoT-based task offloading and optimization of resource utilization and energy efficiency. Flower framework is a scalable Federated Learning framework that supports decentralized model training, real-time updates, and privacy. It integrates with various ML libraries and offers modular components for secure, scalable learning.

### 5.1. Ablation Study

#### 5.1.1. Comparing the Proposed Method With and Without the Attention Mechanism

Table 6 evaluates the impact of incorporating an attention mechanism into the proposed method. The global accuracy increases from 0.5386 (without attention) to 0.70 (with attention), showing a significant performance gain. The global loss decreases from 0.0054 to 0.0034, indicating that the model learns more effectively with the attention mechanism. Client 1 experiences a substantial boost in accuracy from 0.30 to 0.90, though the loss slightly increases from $4.5385\times 10^{-5}$ to $8.5700\times 10^{-4}$. Client 2 also sees an improvement in accuracy from 0.60 to 0.80, while its loss decreases significantly from $2.7892\times 10^{-4}$ to $1.6920\times 10^{-5}$.

The proposed D4PG-based Federated Learning algorithm is analyzed on different learning rates and its effect on model accuracy. The highest accuracy of 92.86% is achieved with a learning rate of 0.001, suggesting that this value enables optimal convergence. With 0.0001, the model achieves 91.56% accuracy, indicating stable learning but possibly slower convergence. At 0.01, accuracy drops to 90.58%, which may indicate instability or ineffective learning. At 0.1, accuracy is 91.23%, which is slightly better but still lower than the optimal learning rate of 0.001.

#### 5.1.2. Analysis for Different Min Probability Thresholds for Client Selection and Noise Levels

**Figure 3:**
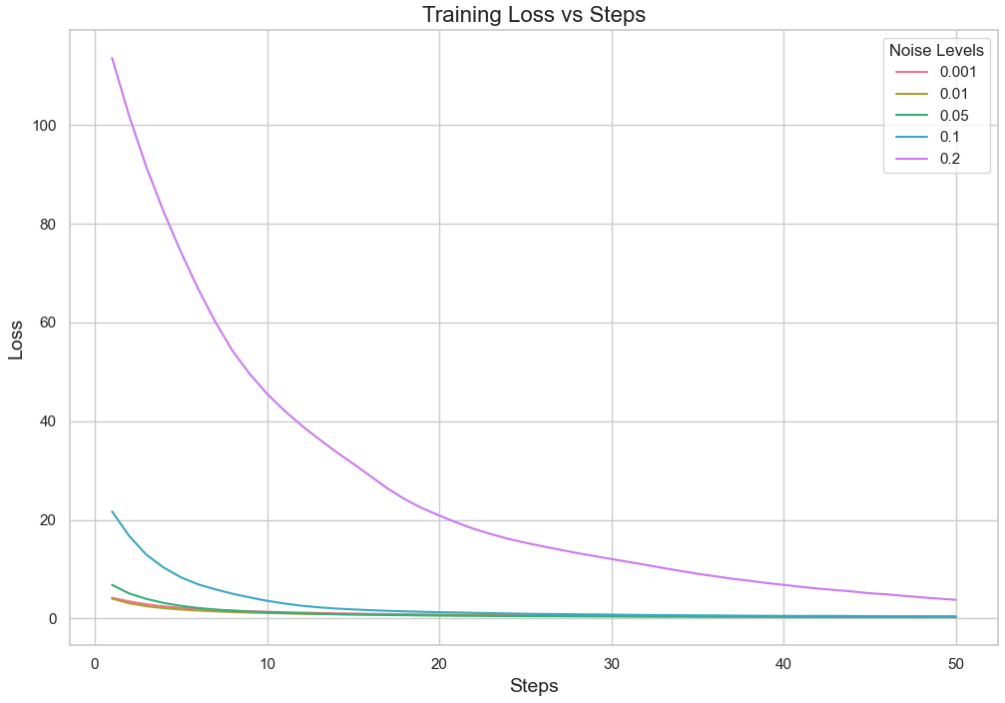
Noise level loss.

**Figure 4:**
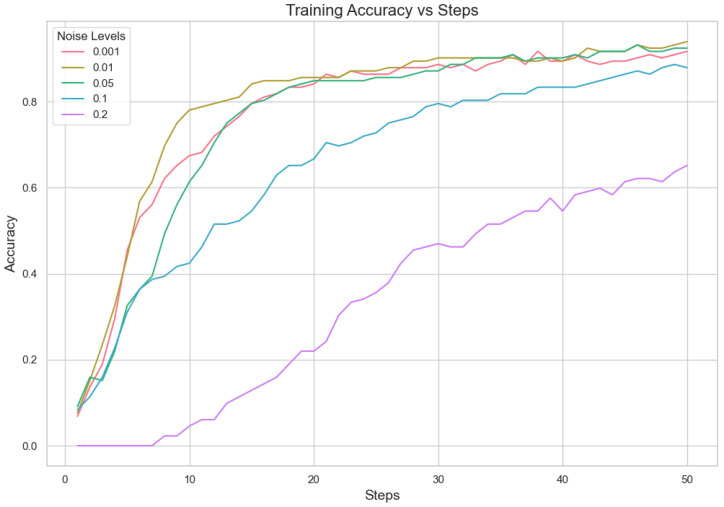
Noise level accuracy.

In our proposed method, we aim to minimize client repetition frequency while varying noise levels to enhance the robustness and generalization of the model. Performance indicator analysis from Table 7 and Figure 3 and Figure 4 shows that a lower Min Probability Threshold offers superior validation loss and correctness under less noisy conditions, specifically at 0.01. However, when there is more noise, model performance decreases notably especially with higher thresholds applied. This means keeping a lower frequency of choosing clients not only supports having different types of clients but also assists in lessening the bad effects that noise might have on how well the model works. In essence, our method highlights how important it is to find a good mix between diversity in picking clients and managing noise. It suggests that aiming for lower repeat frequencies can result in more dependable results under differing working situations. This capacity to adapt is essential for strong performance in practical applications where client behaviors and data quality can often vary.

### 5.2. Performance Comparison of Prediction Models

The server receives updates from clients after they have trained their local models during the fit round. A fit round consists of 100 global iterations with 20 local iterations. In this case, it received three results; this implies that three out of ten sampled clients took part in this round. The server reports details of the training progress, such as loss and accuracy metrics. In the round of evaluation, the server assesses the global model’s performance on data from sampled clients. Here, it sampled two out of ten clients. The log shows that the results from two clients have been received by the server. The check-up gives an idea about how well the global model can be applied to the data of various clients.

The method proposed in Table 8 produces the best outcomes among all models because it achieved a maximum Train Accuracy of 0.70, which implies a successful learning process. Also, this model produced a minimum Train Loss value of only 0.0034, showing efficient training. This same model also offers the highest Eval Accuracy result for Client 1 with a score of 0.90 and a notable Eval Accuracy result of about 0.80 for Client 2. However, the greater Eval Loss for Client 1 ($8.5700\times 10^{-4}$) shows some variation in performance. The MILSTM-CEFL model also works well, with a high Eval Accuracy (0.80) for Client 1 and very low Eval Loss for Client 2 ($3.8304\times 10^{-6}$), showing accurate predictions. In general, including attention mechanisms seems to improve model performance greatly, as shown by our proposed method.

### 5.3. Performance Comparison of Offloading Algorithms

In our study, we tested four offloading algorithms: Worst Fit, Best Fit, First Fit, and the Hybrid Offloading algorithm. We looked into how well these methods work for keeping power usage stable across various edge servers (Table 9). For analyzing the different algorithms, we used consistency indicators, power efficiency rate changes, and other fluctuations related to resource usage and observations specific to each algorithm.

#### 5.3.1. Consistency

In terms of power consumption stability, the Hybrid Offloading Algorithm seems to be more consistent. Both EdgeServer_1 and EdgeServer_2 maintain a uniform power usage of 265 units in every step and algorithm. This implies that these servers can handle steady workloads with dependability. Moreover, EdgeServer_6 has a steady use of nearly 177.18 units for all algorithms apart from Best Fit. In this case, we can observe slight ups and downs up to approximately 200 units for the Best Fit algorithm. This suggests that hybrid offloading and first fit are more stable in handling load on EdgeServer_6 when compared against the best-fit method.

#### 5.3.2. Power Efficiency

Regarding power efficiency, EdgeServer_5 generally has the least power consumption. It uses an average of around 74.51 units across all algorithms, showing occasional spikes in use but mostly staying at this level. A steady pattern of low usage indicates good workload management, particularly with the Hybrid Offloading and First Fit algorithms. The Hybrid Offloading Algorithm is very steady for all servers, with little variation. This makes it the best in terms of power efficiency overall. The First Fit Algorithm is almost as stable, but EdgeServer_4 always shows a bit higher power usage. The Worst Fit Algorithm is not as efficient. It displays more noticeable changes, particularly for EdgeServer_3—it has the highest power consumption when busy and lowest when idle among all servers; the case of EdgeServer_4 and 5 shows similar behavior but not to the same extent as the previous one. The Best Fit Algorithm appears to be mostly steady. However, several times it shows some unsteadiness with swings of up to 200 units in the case of EdgeServer_6.

#### 5.3.3. Fluctuations

The power consumption differences are very small with the Hybrid Offloading Algorithm, keeping the performance stable for all servers. The First Fit Algorithm is similar and shows only slightly more variance, especially on EdgeServer_4. The Worst Fit Algorithm displays large fluctuation in power consumption, particularly on EdgeServer_3, EdgeServer_4, and EdgeServer_5. This is not as predictable and might be an inefficient distribution of workload. The Best Fit Algorithm has some ups and downs, particularly in EdgeServer_6, where power consumption increases to 200 units. It implies that there are fluctuations in how efficiently servers handle their loads.

#### 5.3.4. Algorithm-Specific Observations

The power usage in all servers using the Hybrid Offloading Algorithm is stable and effective, with small fluctuations. It works well for situations needing steady performance. The First Fit Algorithm also shows efficient work, but only slightly less stability than the Hybrid Offloading Algorithm. The Worst Fit Algorithm displays the greatest variety and potential inefficiencies, particularly in handling workloads of EdgeServer_5. At last, even if generally stable, the Best Fit Algorithm does have a few inefficiencies, such as significant alterations in specific servers like EdgeServer_6, suggesting an enhancement requirement for workload management.

### 5.4. Performance Comparison of Federated Learning Algorithms

**Figure 5:**
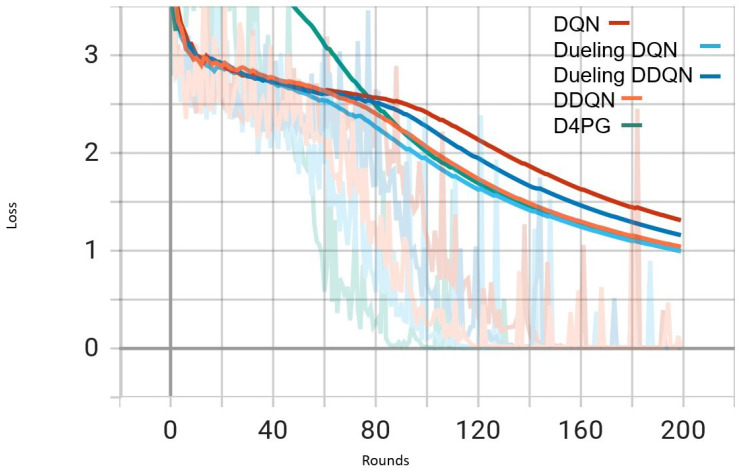
Comparison of loss: DQN, DDQN, Dueling DQN, Dueling DDQN, and D4PG Models on IID EMNIST Dataset.

**Figure 6:**
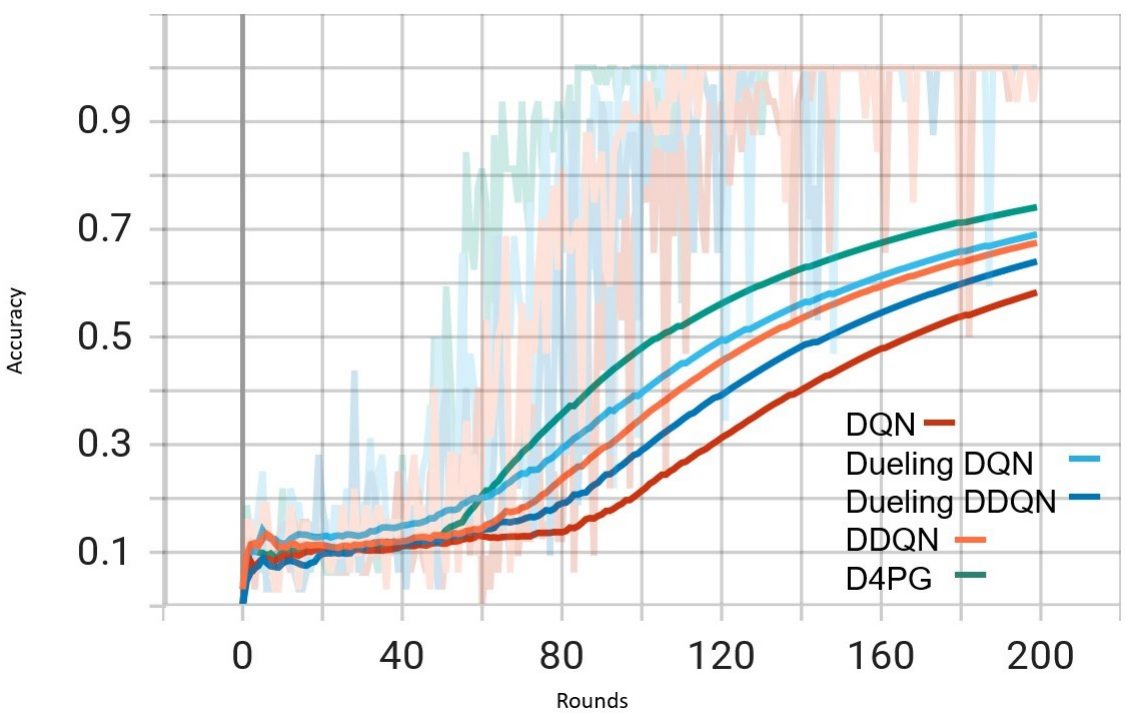
Comparison of accuracy: DQN, DDQN, Dueling DQN, Dueling DDQN, and D4PG Models on IID EMNIST Dataset.

**Figure 7:**
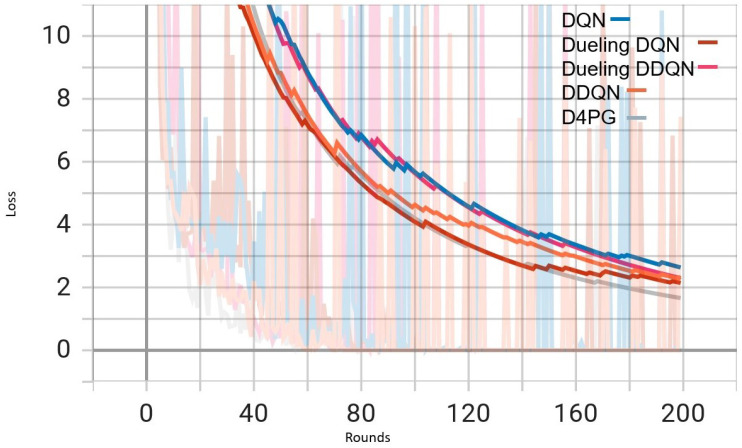
Comparison of loss: DQN, DDQN, Dueling DQN, Dueling DDQN, and D4PG models on Non IID EMNIST Dataset.

**Figure 8:**
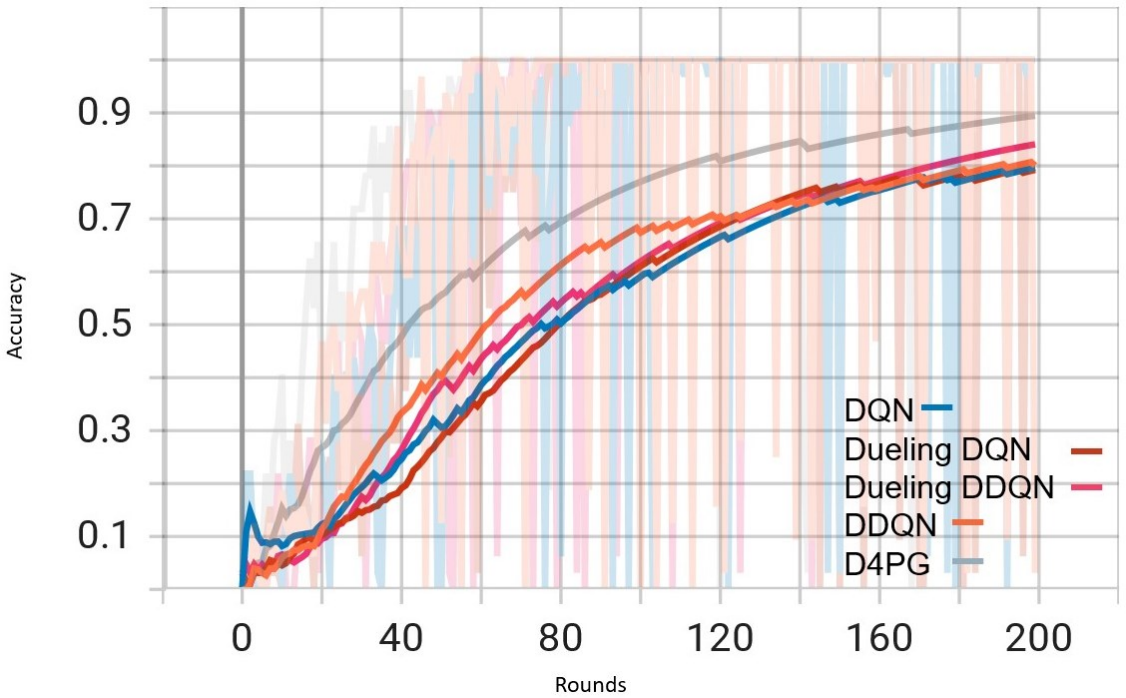
Comparison of accuracy: DQN, DDQN, Dueling DQN, Dueling DDQN, and D4PG models on Non IID EMNIST Dataset.

Our experiment is based on the EMNIST [43] dataset and the Crop Prediction dataset provided publicly by the Indian Chamber of Food and Agriculture (ICFA) under the license CC BY 3.0 IGO. The parameters for the model training are carried out as given in Table 10. We combine D4PG and Federated Learning in a model we propose, using decentralized training together with enhanced value estimation. In every iteration of Federated Learning, client devices calculate local updates while preserving the privacy of data. Data privacy in this model is maintained by incorporating differential privacy via the addition of Gaussian noise to the model weights during each iteration of Federated Learning as in Equation (46). Table 7 presents the impact of different noise levels on model performance, showing how higher noise levels preserve privacy but lead to a decrease in model accuracy. In Federated Learning, the Min Probability Threshold controls client selection, which also influences how noise and data are aggregated. Thus, maintaining privacy involves finding an optimal balance between noise (for privacy) and performance (accuracy) [44]. These updates are aggregated by a central server using D4PG. The training curves in Figure 5, Figure 6, Figure 7 and Figure 8 were smoothed using a parameter of 0.99 in TensorBoard. This high level of smoothing was chosen to emphasize long-term trends and reduce the visual impact of short-term fluctuations.

In Figure 5, all the algorithms have a high loss value at first, but DQN and DDQN are showing a steady decrease. D4PG is performing the best as it reaches the lowest loss around 80 rounds. The rest of the algorithms converge at a slower pace, but they keep reducing their loss and come close to each other around 160 rounds.

In Figure 6, D4PG remains at the topmost position with an overall accuracy higher than 0.8. DQN and DDQN display similar patterns of accuracy improvement, reaching a steady state around 0.75 as iterations proceed further. Every algorithm shows a clear increase in accuracy, and D4PG maintains its lead during the entire training process.

Figure 7 shows that all the algorithms begin with a high loss of around 2.4. Over time, these loss values drop and become less significant as training continues. It is interesting to observe that D4PG experiences a swift and consistent decrease in its loss compared to other algorithms. Around 50 rounds, D4PG’s loss stabilizes more rapidly and at a lesser value compared to the others. It shows its better performance in reducing loss soon after starting the training process. At the end of 200 rounds, D4PG keeps a lower loss value near 0.35 versus other algorithms which converge around 0.4.

In Figure 8, D4PG exhibits a better performance trajectory as well. It starts at low accuracy, but then quickly improves as it reaches higher values faster than other methods. After about 50 rounds, the accuracy of D4PG becomes better than other algorithms and keeps growing at a regular pace. When we finish 200 rounds, D4PG reaches the topmost accuracy with around 0.85 while other algorithms settle near 0.8; this implies that D4PG not only learns quicker but also attains greater final precision.

#### 5.4.1. Analysis Between IID and Non-IID Datasets

Table 11 displays performance metrics of five varying Reinforcement Learning models, namely DQN, DDQN, Dueling DQN, Dueling DDQN, and D4PG when applied to the Non-IID EMNIST dataset. The values for performance metrics are shown in decimal numbers ranging from zero to one. DQN has the poorest performance with recall, F1 score, and accuracy of just 0.75, but it also has the highest loss at 0.84. For DDQN, we see an improvement with recall, F1 score, and accuracy, all at 0.82; loss is down to only being as high as 0.64. Dueling DQN shows nearly the same scores as DDQN, but it has a better loss of 0.60. For Dueling DDQN, the metrics are a little lower, with recall at 0.79 and loss being 0.77. D4PG performs well with a recall of 0.83 and a loss of 0.62.

According to the table with results from different models on IID EMNIST in Table 12, the D4PG model has top recall and precision at 0.82 and 0.83, respectively, showing its outstanding ability to correctly find positive instances and keep false positives low. Additionally, it also displays the highest accuracy score of 0.82 which shows how well-rounded this model is in correctly classifying cases overall. The F1 score is at 0.82, showing the balance between precision and recall. We can see that the D4PG model has the lowest loss value of all the models listed here, 0.61. This low loss shows that the model is converging well and strongly. Together, these metrics confirm D4PG as superior; it outperforms other models, which makes D4PG the most dependable and efficient model for this task on the IID EMNIST dataset.

#### 5.4.2. Analysis with High Number of Classes in Non-IID EMNIST Dataset

**Figure 9:**
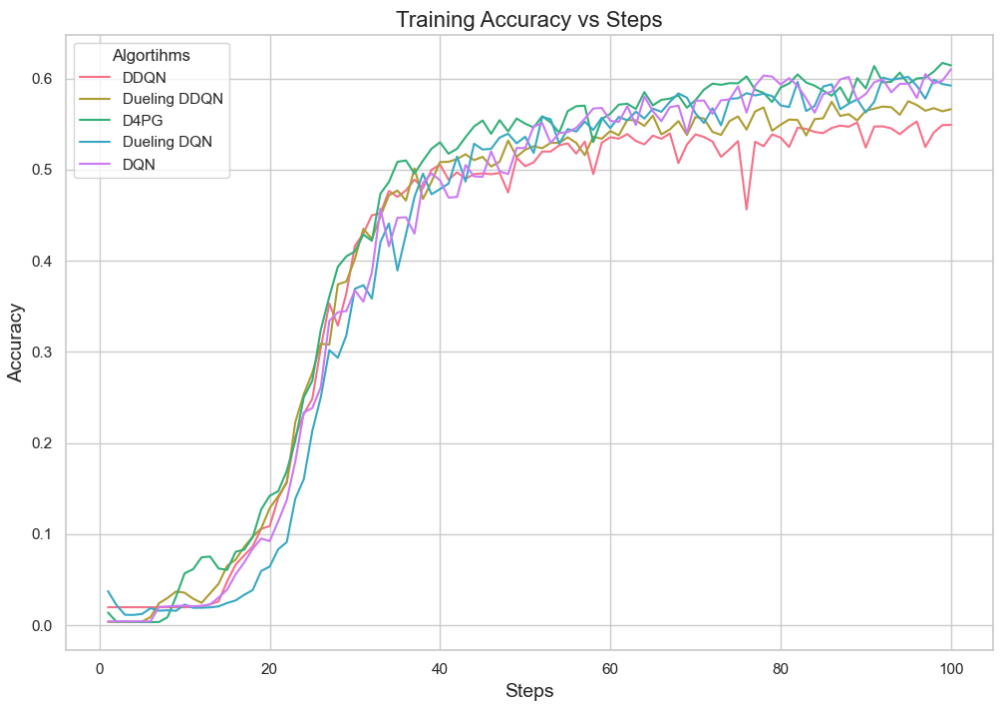
Comparison of accuracy: DQN, DDQN, Dueling DQN, Dueling DDQN, and D4PG models on 62 classes Non-IID EMNIST Dataset.

**Figure 10:**
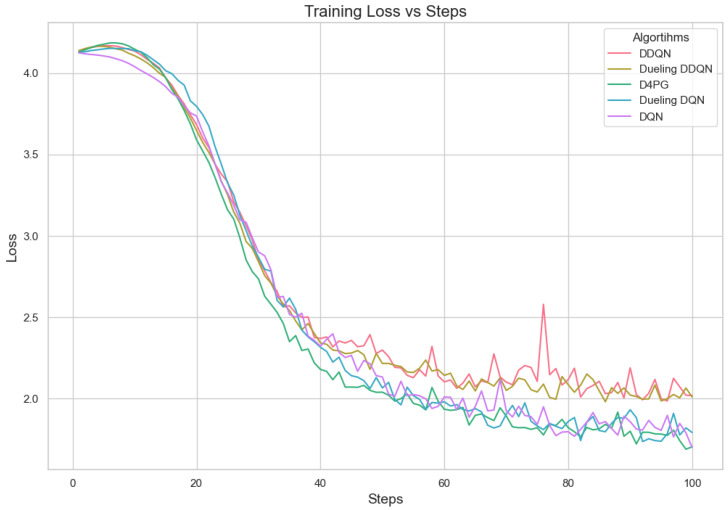
Comparison of loss: DQN, DDQN, Dueling DQN, Dueling DDQN, and D4PG models on 62 classes Non-IID EMNIST Dataset.

The analysis of Deep Reinforcement Learning models—DQN, DDQN, Dueling DQN, Dueling DDQN, and D4PG—on the 62 classes of the Non-IID EMNIST Dataset reveals distinct performance characteristics across various metrics. The accuracy versus training steps graph Figure 9 shows that all models initially improve rapidly, but D4PG consistently achieves the highest accuracy as training progresses. Also, the loss graph Figure 10 shows the consistent decrease in model losses in the D4PG model. This observation aligns with the performance metrics Table 13, where D4PG demonstrates superior results with a recall of 0.6106, precision of 0.5811, F1 score of 0.5754, and accuracy of 0.6106 while also maintaining the lowest loss value of 1.6810. These results highlight D4PG’s robustness and adaptability, likely due to its ability to manage continuous actions effectively, making it well-suited for complex, non-IID datasets. In contrast, DDQN and Dueling DDQN exhibit slightly less robust performance, with DDQN achieving a recall and accuracy of 0.5569 and a higher loss of 1.9808. Dueling DDQN also fails to significantly outperform its basic counterpart, suggesting that the benefits of the dueling architecture are not fully realized in this scenario. Meanwhile, Dueling DQN shows better performance than DDQN, with an accuracy of 0.5842 and a loss of 1.8222, indicating some advantage of the dueling architecture, although this advantage is context-dependent and may require further optimization. DQN, the simplest model, serves as a benchmark, showing reasonable recall and precision but higher loss (1.7372), indicating less effective optimization compared to D4PG. This analysis underscores the importance of advanced techniques such as prioritized replay and continuous action handling, as demonstrated by D4PG, in enhancing learning outcomes in challenging Federated Learning contexts. Overall, the differences in model performance emphasize the critical role of architectural choices in effectively addressing complex data scenarios.

#### 5.4.3. Analysis of Different Number of Clients

**Figure 11:**
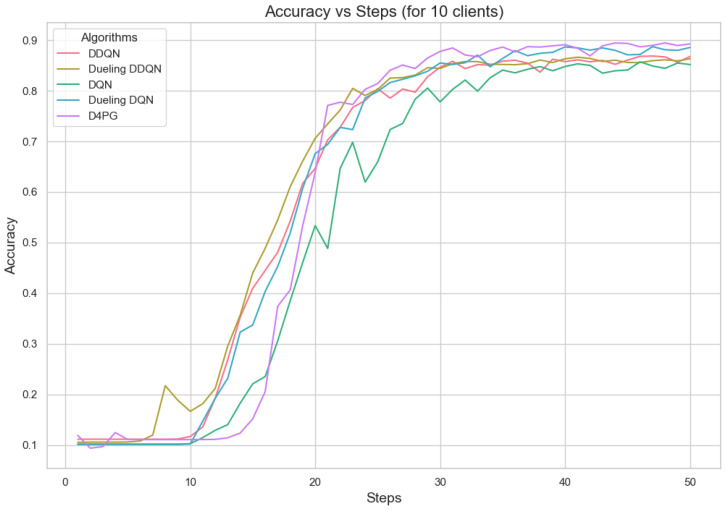
Accuracy for 10 clients.

**Figure 12:**
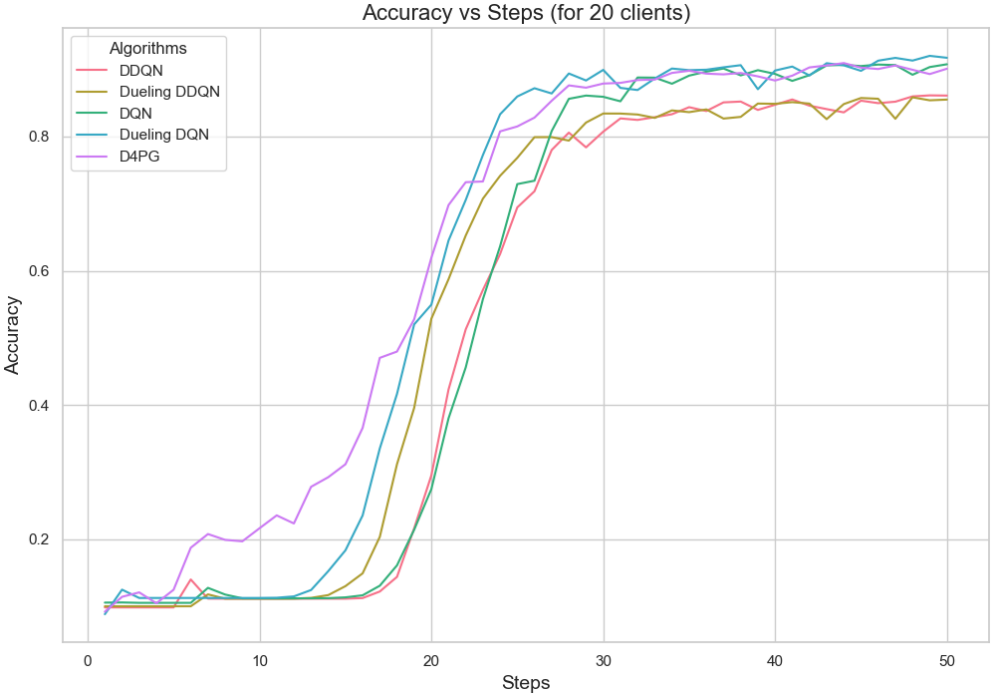
Accuracy for 20 clients.

**Figure 13:**
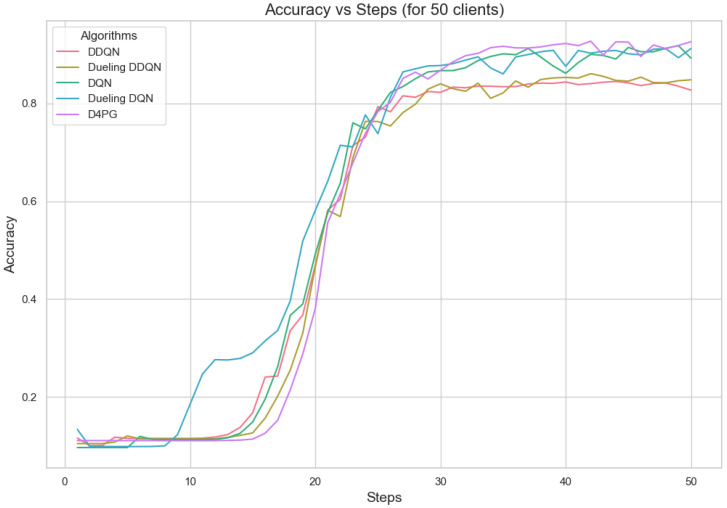
Accuracy for 50 clients.

**Figure 14:**
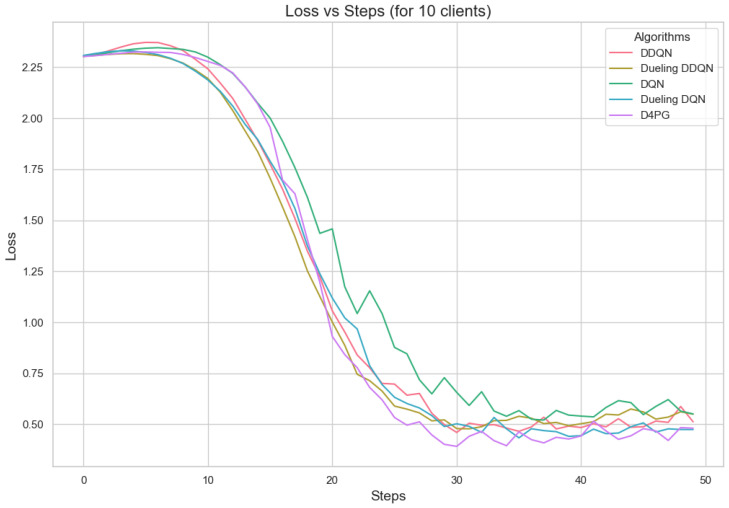
Loss for 10 clients.

**Figure 15:**
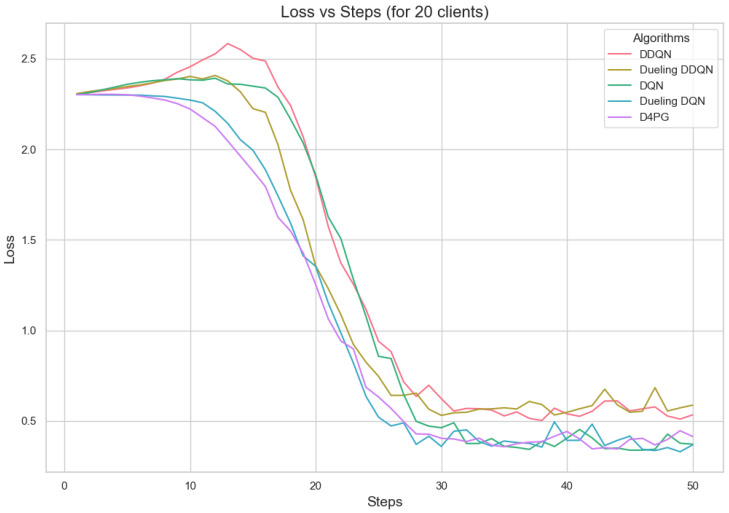
Loss for 20 clients.

**Figure 16:**
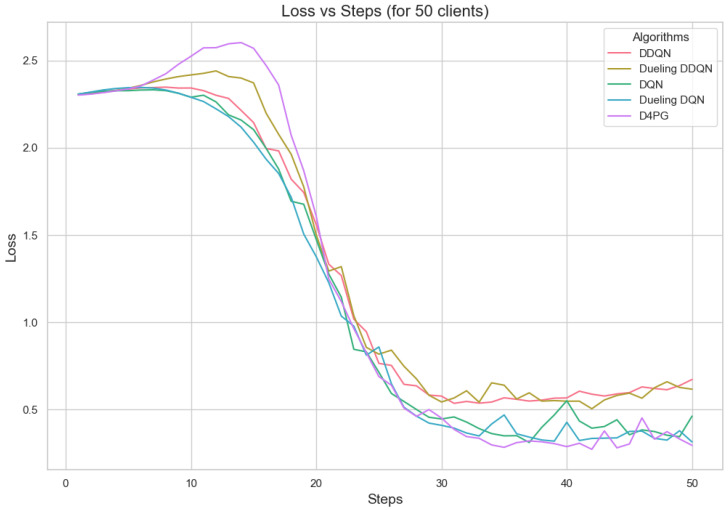
Loss for 50 clients.

Figure 11, Figure 12, Figure 13, Figure 14, Figure 15 and Figure 16 show the analysis of training accuracy and training loss in different numbers of clients. The analysis of the performance metrics (Table 14) for the DQN, DDQN, Dueling DQN, Dueling DDQN, and D4PG models on the Non-IID EMNIST dataset with varying numbers of clients (10, 20, and 50) reveals significant insights into how these models handle Federated Learning scenarios with differing data distributions. The DQN model shows impressive performance with 10 and 20 clients, achieving high accuracy and low loss, indicating its robustness in dealing with smaller client pools. However, its performance slightly declines with 50 clients, suggesting challenges in managing increased data heterogeneity. The DDQN model performs reasonably well with 10 and 20 clients but struggles with 50 clients, showing a noticeable drop in accuracy and an increase in loss, highlighting its limitations in scaling effectively with more clients. The Dueling DQN model consistently performs well across all client numbers, especially excelling with 50 clients, where it achieves high accuracy and the lowest loss among all models. This demonstrates its ability to leverage larger, diverse datasets effectively. Conversely, the Dueling DDQN model starts strong but shows a slight dip in performance with 20 clients, stabilizing with 50 clients but not achieving the same level of effectiveness as the Dueling DQN model. The D4PG model emerges as the most adaptable across different client sizes, maintaining strong performance with 10 clients and achieving top results with 20 and 50 clients, where it records the highest accuracy and minimal loss. This suggests that the D4PG model is particularly well suited for Federated Learning environments, effectively handling the challenges posed by non-IID data distributions across a large number of clients. In summary, while all models have their strengths, the D4PG and Dueling DQN models stand out for their robustness and adaptability, making them ideal choices for Federated Learning applications that require managing diverse and large datasets.

#### 5.4.4. Analysis with Crop Prediction Dataset

**Figure 17:**
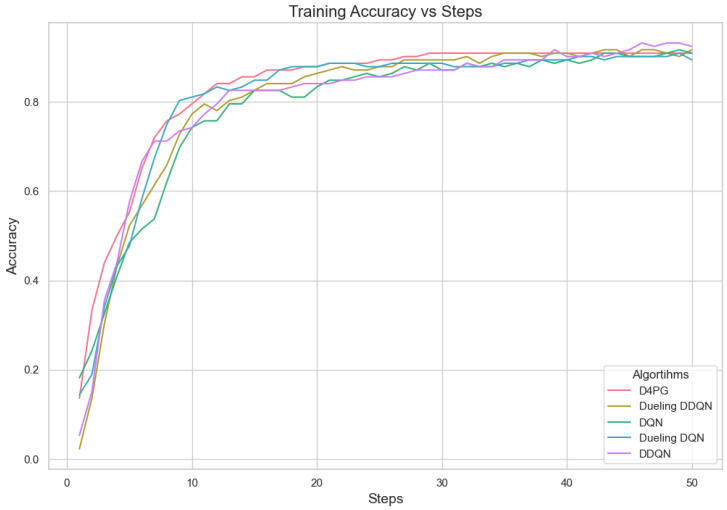
Training accuracy for crop prediction dataset.

**Figure 18:**
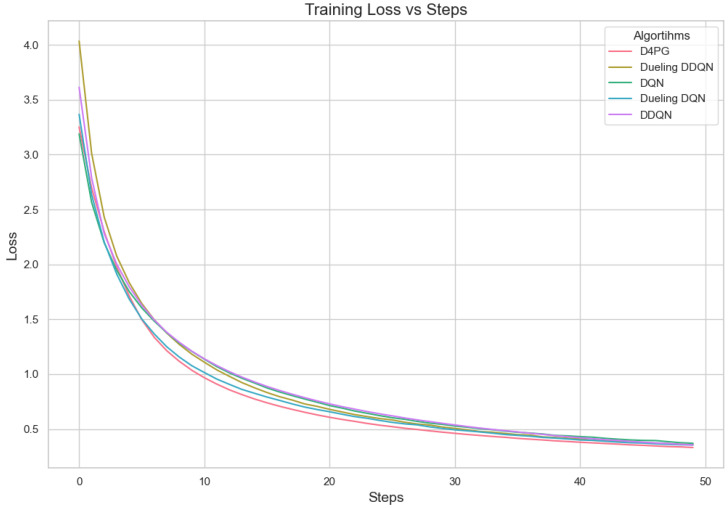
Training loss for crop prediction dataset.

Further, we tested the proposed model on the Crop prediction dataset. The Figure 17 and Figure 18 show the model training accuracy and training loss in the crop prediction dataset. In these models that were tested, it is shown (Table 15) that D4PG has the best performance with a 92.86% accuracy rate and the highest weighted average F1-score of 0.93. This shows that D4PG not only has the greatest total accuracy, but it also keeps up its performance in different classes.

Dueling DDQN and DQN perform well, too, having accuracies of 91.88% and 91.56%, respectively, with weighted average F1-scores of about 0.92 and 0.91. This shows that these models are also dependable but a bit less effective than the D4PG model. DDQN performs well: it has an accuracy of 90.91% and a weighted average F1-score of about 0.91.

On the other hand, the Dueling DQN model has the worst performance among all tested models. It shows an accuracy rate of 89.29% and a weighted average F1-score of 0.88. The drops in performance for some classes are noticeable, especially class number 13, which affects its overall functioning power but still holds relatively high accuracy and performance in other classes, too.

#### 5.4.5. Analysis of the Reward per Episode Graph

**Figure 19:**
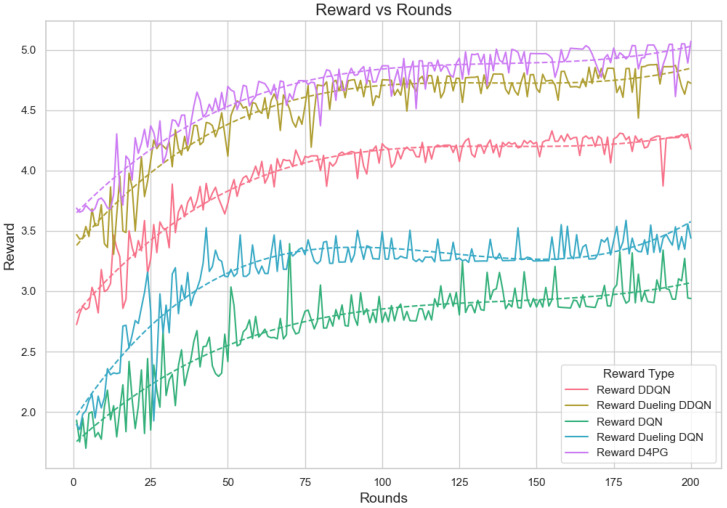
Analysis of the Reward Per Episode Graph for different Reinforcement Learning algorithms in Federated Learning setup.

The reward is computed out of a combined reward score that takes into account fairness, resource usage, and accuracy. The fairness part of the reward is calculated by obtaining the class distribution data. Next, it uses these data to find out how fairly resources or outcomes are distributed among different classes. The resource reward uses the average of resources used, showing how efficient the use of all resources is. After calculating these two rewards, they are added together with the accuracy score. Each is multiplied by its weight. The total reward comes from adding up these weighted parts, which gives us an even measurement for performance, taking into account fairness, usage of resources, and accuracy in prediction results. Figure 19 displays reward per episode for 200 episodes and shows the learning process of different Reinforcement Learning algorithms in a Federated Learning setting.

D4PG and Dueling DDQN present the best and steady rewards that show they are superior in performance and learning capability. The graph shows that the D4PG algorithm has the maximum rewards. It starts slightly above 3.0 and increases consistently to about 4.8, which implies strong learning performance with moderate volatility. The Dueling DDQN algorithm also experiences significant improvement. It starts slightly below 3.5 and gradually increases to approximately 4.5, indicating effective learning over time with a bit more oscillations compared to D4PG. The DDQN demonstrates an obvious increase from below 3.0 up until just above 4.0, indicating good performance with moderate stability.

The rewards of DQN and Dueling DQN are lower with more fluctuations, indicating that learning and stability are not as effective. The DQN and Dueling DQN algorithms have a beginning point with lower rewards around 2.0 and more turbulent behavior. DQN increases to about 3.0 while the Dueling DQN reaches nearly 3.5; both show considerable changes, suggesting unstable learning occurs. The polynomial trend lines support these observations, pointing out the overall trends and consistent performance of every algorithm across rounds.

**Figure 20:**
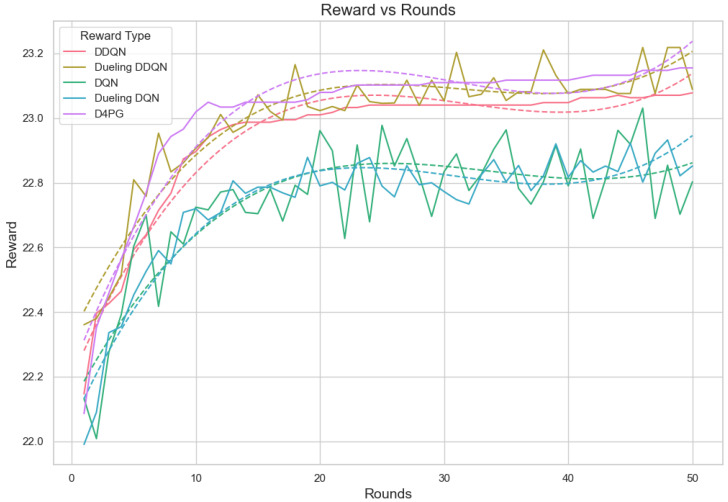
Analysis of the Reward Per Episode Graph for different Reinforcement Learning algorithms in Federated Learning setup for crop prediction dataset.

In the evaluation of reward per episode for Crop Prediction Dataset Figure 20, the Dueling DDQN algorithm is the best. It reaches an episode maximum of 23.12 and the topmost value of reward per step at 0.463, showing the increased capability of gathering rewards compared to other algorithms in both the overall performance and on a per-step basis. The D4PG algorithm is the next best, with an episode reward of 23.02 and a reward per step value of 0.460, performing quite impressively. The DDQN algorithm is performing quite well. It has an episode reward of 22.55 and a reward per step of 0.452, thus being efficient in gaining rewards. The DQN algorithm seems to be less efficient in this area, with lower numbers both for episode reward 21.78 and the lowest reward every step 0.436. This points to its relative inefficiency compared to the other two methods. On average, Dueling DDQN reaches the top performance among these models, but also shows slightly worse performance than average sometimes, too, because its best model does not always perform equally well.

## 6. Discussion and Conclusions

This study presents a robust framework for efficient resource management and task scheduling in Agricultural IoT by integrating machine learning-based forecasting with Federated Reinforcement Learning. The proposed hybrid BiLSTM-GRU with the attention mechanism model significantly enhances resource usage prediction, enabling adaptive task scheduling that minimizes energy consumption across edge servers. The ability of our model to optimize resource allocation and task offloading in real time ensures that the system can handle an increasing number of devices without compromising performance. This approach contributes to optimizing resource utilization, offering a promising solution for dynamic task distribution in edge computing environments. The integration of a hybrid offloading algorithm optimizes performance by dynamically distributing tasks based on real-time resource availability. Experimental results validate the effectiveness of this approach, demonstrating notable improvements in scalability, energy efficiency, and system reliability over existing methods. Additionally, we also propose the D4PG based on a Federated Learning algorithm for adjusting the participation of dynamic User Equipment (UE) according to resource availability and data distribution. Among the Reinforcement Learning techniques explored, D4PG outperforms others by reducing loss and increasing accuracy, while the Dueling architectures show exceptional performance during the early stages of learning. While DQN and DDQN exhibit steady performance, they converge more slowly compared to the advanced models. The theoretical insights derived from this research underline the importance of combining machine learning, Reinforcement Learning, and edge computing in solving complex IoT challenges, particularly in dynamic and resource-constrained environments. Furthermore, the proposed hybrid model’s ability to handle non-IID data distributions and ensure privacy preservation in federated settings provides a significant advancement in the field of Federated Learning for IoT applications. Our proposed model is particularly well-suited for Agricultural IoT applications due to its ability to process large-scale time-series data and dynamically optimize resource allocation and task scheduling in real time. In smart farming, IoT devices continuously collect data from various sources, such as soil moisture sensors, weather stations, and crop health monitoring systems. Efficiently managing and analyzing this data is crucial for improving crop yield, minimizing resource waste, and ensuring sustainable farming practices. The D4PG in our framework enhances decision-making by enabling adaptive task scheduling and resource allocation across distributed edge nodes in an agricultural setting. This allows for real-time processing and reduced latency, which is essential for applications like automated irrigation, pest control, and precision agriculture. Additionally, Federated Learning ensures data privacy and security by keeping sensitive farm data localized while collaboratively training global models. Beyond Agricultural IoT, our model has broader applicability in various IoT domains where dynamic resource allocation and intelligent task scheduling are critical. For example, efficient traffic management, energy distribution, and public infrastructure monitoring; predictive maintenance, autonomous manufacturing processes, and real-time monitoring of supply chains; remote patient monitoring, resource allocation in hospitals, and adaptive scheduling of medical IoT devices. By enabling scalable, privacy-preserving, and adaptive resource management, this study contributes to the advancement of intelligent edge computing for next-generation IoT applications, providing a solid foundation for future research and real-world deployment.

From the results, we can corroborate that the scalability of our proposed framework is backed by a Federated Learning framework. This allows decentralized training on multiple devices without needing data transfer to a central server. It lowers communication overhead and lets the system manage an increasing number of networked devices efficiently. The edge computing structure used in our study also aids the distribution of computation tasks, lessening individual device strain and avoiding bottlenecks while more devices join up. The hybrid forecasting model, using BiLSTM and GRU along with the attention mechanism techniques, ensures the prediction of resource use remains accurate even when the network size increases. Edge resource competition, where multiple devices simultaneously request processing power, is a critical challenge in edge computing environments. Our approach addresses this issue by utilizing dynamic task offloading strategies that prioritize tasks based on real-time resource availability and predicted workload. The D4PG Reinforcement Learning model adapts according to changes in resource conditions and manages the involvement of User Equipment (UE) based on the demand-supply balance for resources, thus minimizing competition over resources. Furthermore, using a hybrid prediction model guarantees effective resource allocation. It prevents the overloading of edge servers and balances the resource demands across devices. The proposed architecture uses Federated Learning to enhance coordination among distributed devices without compromising privacy. Federated Learning allows each device to train models locally while only sharing model updates; this ensures coordination between edge devices without centralizing data. The D4PG model offers improvement to this coordination. It ensures each device can adjust its behavior based on global learning. This leads to more efficient task scheduling and resource utilization. Plus, the attention mechanism in the prediction model makes coordination better by letting the system concentrate on relevant features when predicting resource usage. This helps allocate tasks more precisely.

While the approach demonstrates good performance in smaller agricultural IoT environments, the system’s performance might be impacted by the complexity and heterogeneity of large networks. Although the proposed method effectively manages dynamic resource allocation, real-time adaptation to highly unpredictable network conditions, such as unexpected network failures, sudden spikes in task load, or varying edge device availability, may require further optimization. While our approach improves resource efficiency and scheduling, extreme fluctuations in network conditions could introduce delays or suboptimal allocation decisions. Future enhancements could incorporate adaptive learning mechanisms, such as Reinforcement Learning-based optimization or self-adjusting heuristics, to further enhance the system’s responsiveness and robustness in handling highly volatile environments.

In summary, the proposed Federated Reinforcement Learning-based task offloading algorithm optimizes resource utilization, reducing energy consumption while maintaining system performance in edge-based IoT environments. BiLSTM-GRU with the attention model accurately predicts resource usage, enabling dynamic and adaptive scheduling in IoT applications. The D4PG-based Federated Learning approach enhances fairness in model updates while preserving user privacy, which is crucial for distributed IoT systems. Experimental results demonstrate that the proposed method outperforms existing algorithms (best-fit, first-fit, worst-fit) and deep RL baselines (DQN, DDQN, Dueling DQN, Dueling DDQN).

## Figures and Tables

**Table 1:** Summary of Dynamic Resource Allocation and Task Scheduling in Edge for IoT Applications.

Framework	Key Focus	Advantages	Limitations
MLSTM-CEFL [26]	Congestion-aware timing and resource combination	Improves performance in congestion scenarios	Neglects fairness in resource allocation, leading to performance imbalances for some users
BiLSTM [27]	Mobile Edge Computing (MEC) in 5G networks	Handles changing conditions over time	Lacks integration of stochastic models for uncertainty in wireless channels and data volumes
TrustFedGRU [28]	Dynamic update strategies for FL in IIoT	Improves privacy and security	Does not account for non-IID data distributions or varying computational abilities in clients
FedBi-GRU [29]	Virtual Network Functions (VNFs) for local training	Enhances confidentiality and reduces computational burden	Struggles with the dynamic and non-permanent nature of IoT settings, including traffic and resource fluctuations
BiLSTM-GRU [30]	Cloud resource prediction model	Improves prediction precision and reduces time	Does not address fairness in resource distribution, especially in shared cloud environments
FeDRL-D2D [31]	Energy-efficient resource allocation in D2D-assisted HetNets	Focuses on power control and resource allocation for limited devices	Static reward function, lacks scalability for real-world D2D-assisted 6G networks, does not optimize real-time trade-offs
Dueling-DQN [32]	Resource allocation and vehicle participation optimization	High precision in map caching for vehicles	Does not account for non-IID data in real-world driving scenarios; fairness issues in vehicle participation
DQN [33]	Node selection in heterogeneous FL networks	Suitable for networks with data and hardware heterogeneity	Does not adapt to dynamic changes in the network or fluctuating performance across devices
DDQN [34]	Offloading decisions in MEC environments	Efficient offloading using DDQN agents	Does not consider heterogeneous mobile devices in Federated Learning, neglecting device characteristics like battery life and network connectivity

**Table 2:** Notation and Variable Definitions.

Symbol	Description
$\mathrm{Performance}(x_{ij})$	Performance metric for assigning task $t_{i}$ to edge device $d_{j}$
$\mathrm{OffloadCost}(y_{jk})$	Cost of offloading data from edge device $d_{j}$ to the edge server $e_{k}$
λ	Weighting factor to balance performance and offload cost
$\mathrm{DataLoad}(x_{ij})$	represents the data load associated with assigning task $t_{i}$ to edge device $d_{j}$
$\mathrm{DataOffload}(y_{jk})$	represents the data offload from edge device $d_{j}$ to the cloud server $e_{j}$
$\mathrm{Capacity}(d_{j})$	is the capacity constraint for edge device $d_{j}$
*N* as the total number of UEs (user equipments)	
$x_{i}$	represents the learning progress of UE *i*, where i=1,2,…,N
$f(x_{1},x_{2},\ldots ,x_{N})$	represents the objective function representing the learning process, which depends on the progress of all UEs
η	represents the learning rate, determining the step size in the learning process

**Table 3:** Notation and variable Definitions.

Symbol	Description
$\mathrm{PerformanceMetric}(x_{ij})$	is a metric representing the performance of assigning task $t_{i}$ to device $d_{j}$
$\mathrm{EnergyConsumption}(d_{j})$	is the energy consumption of device $d_{j}$
λ	is a weighting factor to balance performance and energy consumption
$\gamma_{e_{j},t}$	signal-to-noise ratio (SNR) for the communication channel between the end user and the edge server for task *t*
$P_{u}$	transmit power from the end user
$h_{e_{j},t}$	channel gain between the end user and the edge server for task *t*
$n_{t}$	noise power at the edge server
$h_{n,t^{\prime },t}$	channel gain between the end user and the edge server for task $t^{\prime }$
$t^{\prime }$	belongs to the set *T* of all tasks except *t*
$\sigma^{2}$	noise power

**Table 4:** Notation and Variable Definitions.

Symbol	Description
$w_{\mathrm{global}}$	denotes the global model parameters
$w_{k}$	denotes the local model parameters for UE *k*
$p_{k}$	denotes the sampling probability for UE *k* in $K_{\mathrm{tot}}$
λ	denotes the regularization strength
$K_{\mathrm{sampled}}$	is the size of the sampled subset of UEs
$L_{k}\left(w_{k}\right)$	is the local loss function for UE *k* with model parameters $w_{k}$
$\left|\right|w_{k}-w_{\mathrm{global}}{\left|\right|}^{2}$	is the squared Euclidean distance between the local model parameters and the global model parameters

**Table 5:** Architecture Details for the BiLSTM-GRU Model with attention mechanism.

Name of Parameter	Details
Input layer neurons	43 (depends on input feature dimension)
BiLSTM layer neurons	512 in each direction (total 1024 combined)
Attention layer	Custom attention layer
GRU layer neurons	128
Final layer (Dense) neurons	2
Training step/epochs	[20–100]
Batch size	[16–256]
Optimizer	adam
Activation function	ReLU/Tanh/Sigmoid
Loss function	Mean squared error

**Table 6:** Comparing The proposed method with and without the attention mechanism.

Model	Train Accuracy	Train Loss	Eval Accuracy (Client 1)	Eval Loss (Client 1)	Eval Accuracy (Client 2)	Eval Loss (Client 2)
Without Attention Mechanism	0.54	0.0054	0.30	$4.5385\times 10^{-5}$	0.60	$2.7892\times 10^{-4}$
With Attention Mechanism	0.70	0.0034	0.90	$8.5700\times 10^{-4}$	0.80	$1.6920\times 10^{-5}$

**Table 7:** Performance metrics for different Min Probability Thresholds and Noise Levels.

Min Probability Threshold	Noise Level	Val Loss	Val Accuracy
0.01	0.001	0.250866	0.928939
	0.01	0.248658	0.927273
	0.05	0.195635	0.935303
	0.1	0.38984	0.901212
	0.2	2.405279	0.831515
0.05	0.001	0.250337	0.932879
	0.01	0.258329	0.927273
	0.05	0.24644	0.921667
	0.1	0.352063	0.915303
	0.2	2.761476	0.801364
0.1	0.001	0.909531	0.801667
	0.01	0.950542	0.781667
	0.05	1.153836	0.764848
	0.1	3.187835	0.635303
	0.2	19.31784	0.423636

**Table 8:** Performance Metrics for Prediction Models.

Model	Train Accuracy	Train Loss	Eval Accuracy (Client 1)	Eval Loss (Client 1)	Eval Accuracy (Client 2)	Eval Loss (Client 2)
TrustFedGRU [28]	0.63	0.0062	0.80	$2.0606\times 10^{-4}$	0.70	$2.1974\times 10^{-5}$
FedBi-GRU [29]	0.55	0.0048	0.60	$1.8800\times 10^{-4}$	0.50	0.0545
MILSTM-CEFL [26]	0.67	0.0069	0.80	$2.4292\times 10^{-5}$	0.70	$3.8304\times 10^{-6}$
BiLSTM [27]	0.55	0.0056	0.60	$5.9681\times 10^{-5}$	0.70	$4.4949\times 10^{-6}$
BiLSTM-GRU [30]	0.68	0.0054	0.80	$2.0387\times 10^{-5}$	0.60	$1.6965\times 10^{-5}$
Proposed Method	0.70	0.0034	0.90	$8.5700\times 10^{-4}$	0.80	$1.6920\times 10^{-5}$

**Table 9:** Showing the standard deviation of power consumption for each edge server with Hybrid Offloading, First Fit, Best Fit, and Worst Fit algorithms.

Edge Server	Algorithm			
	Hybrid	First	Worst Fit	Best Fit
EdgeServer_1	0	0	0	0
EdgeServer_2	0	0	0	0
EdgeServer_3	24.484	24.756	25.693	24.484
EdgeServer_4	24.485	24.756	25.693	26.291
EdgeServer_5	12.533	20.489	19.104	5.933
EdgeServer_6	0	0	0	10.449

**Table 10:** Parameter Settings for Training DQN, DDQN, Dueling DQN, Dueling DDQN, and D4PG Models.

Parameter	Non-IID EMNIST	IID EMNIST	Crop Prediction
G (Global rounds)	200	200	50
NUM_CLIENTS	10	10	10
BATCH_SIZE	32	32	32
L (Local training iterations)	20	20	10
LR (Learning rate)	0.05	0.05	0.001
MU (Regularization Strength)	0.01	0.01	0.01
EPSILON (Exploration rate)	0.1	0.1	0.1
TARGET_UPDATE_INTERVAL	5	5	5
MAX_BUFFER_SIZE	10,000	10,000	10,000
ALPHA (Priority exponent)	0.6	0.6	0.6
BETA (Importance sampling exponent)	0.4	0.4	0.4
GAMMA (Discount factor for future rewards)	0.99	0.99	0.99

**Table 11:** Performance Metrics for DQN, DDQN, Dueling DQN, Dueling DDQN, and D4PG Models on the Non-IID EMNIST Dataset.

Model	Recall	Precision	F1 Score	Accuracy	Loss
DDQN [31]	0.8689	0.8722	0.8689	0.8689	0.50897
Dueling DDQN [34]	0.8675	0.8693	0.8668	0.8675	0.53662
DQN [33]	0.8885	0.8945	0.8888	0.8885	0.47522
Dueling DQN [32]	0.8852	0.8898	0.8847	0.8852	0.47577
Proposed(D4PG)	0.8918	0.8941	0.8916	0.8918	0.43822

**Table 12:** Performance Metrics for DQN, DDQN, Dueling DQN, Dueling DDQN, and D4PG Models on the IID EMNIST Dataset.

Model	Recall	Precision	F1 Score	Accuracy	Loss
DQN	0.76	0.77	0.75	0.76	0.86
DDQN	0.80	0.80	0.80	0.80	0.70
Dueling DQN	0.77	0.78	0.76	0.77	0.79
Dueling DDQN	0.76	0.78	0.75	0.76	0.88
Proposed (D4PG)	0.82	0.83	0.82	0.82	0.61

**Table 13:** Performance Metrics for DQN, DDQN, Dueling DQN, Dueling DDQN, and D4PG Models on the 62 classes Non-IID EMNIST Dataset.

Model	Recall	Precision	F1 Score	Accuracy	Loss
DQN	0.6061	0.5659	0.5659	0.6061	1.7372
DDQN	0.5569	0.5386	0.5210	0.5569	1.9808
Dueling DQN	0.5842	0.5603	0.5496	0.5842	1.8222
Dueling DDQN	0.5640	0.5438	0.5377	0.5640	2.0092
Proposed(D4PG)	0.6106	0.5811	0.5754	0.6106	1.6810

**Table 14:** Performance Metrics for DQN, DDQN, Dueling DQN, Dueling DDQN, and D4PG Models on the different number of clients Non-IID EMNIST Dataset.

Model	Client	Recall	Precision	F1 Score	Accuracy	Loss
DDQN	10	0.8689	0.8722	0.8689	0.8689	0.50897
DDQN	20	0.8692	0.8710	0.8687	0.8692	0.50758
DDQN	50	0.8285	0.8568	0.8336	0.8285	0.64657
Dueling DDQN	10	0.8675	0.8693	0.8668	0.8675	0.53662
Dueling DDQN	20	0.8505	0.8542	0.8504	0.8505	0.60313
Dueling DDQN	50	0.8494	0.8532	0.8486	0.8494	0.61122
DQN	10	0.8885	0.8945	0.8888	0.8885	0.47522
DQN	20	0.9026	0.9043	0.9019	0.9026	0.40192
DQN	50	0.8788	0.8907	0.8769	0.8788	0.53618
Dueling DQN	10	0.8852	0.8898	0.8847	0.8852	0.47577
Dueling DQN	20	0.9001	0.9121	0.9023	0.9001	0.40195
Dueling DQN	50	0.9161	0.9195	0.9164	0.9161	0.30606
D4PG (proposed)	10	0.8918	0.8941	0.8916	0.8918	0.43822
D4PG (proposed)	20	0.9095	0.9149	0.9095	0.9095	0.40091
D4PG (proposed)	50	0.9192	0.9223	0.9192	0.9192	0.32927

**Table 15:** Performance Metrics for DQN, DDQN, Dueling DQN, Dueling DDQN, and D4PG Models on Crop Prediction Dataset.

Model	Accuracy	Macro Avg			Weighted Avg	
		Precision	Recall	F1-Score	Precision	Recall
DDQN	90.91%	0.91	0.91	0.90	0.92	0.91
Dueling DDQN	91.88%	0.92	0.93	0.92	0.92	0.92
DQN	91.56%	0.92	0.92	0.91	0.92	0.92
Dueling DQN	89.29%	0.90	0.90	0.89	0.90	0.89
Proposed(D4PG)	92.86%	0.93	0.93	0.93	0.93	0.93

## Data Availability

The datasets generated and/or analyzed and coded on simulations during the current study can be found online.
